# Expression profiling analysis reveals key microRNA–mRNA interactions in patients with transposition of the great arteries and systemic left and right ventricles

**DOI:** 10.3389/fcvm.2022.1056427

**Published:** 2023-01-12

**Authors:** Masood Abu-Halima, Viktoria Wagner, Shusruto Rishik, Tanja Raedle-Hurst, Eckart Meese, Hashim Abdul-Khaliq

**Affiliations:** ^1^Institute of Human Genetics, Saarland University Medical Center, Homburg, Germany; ^2^Department of Paediatric Cardiology, Saarland University Medical Center, Homburg, Germany; ^3^Center for Clinical Bioinformatics, Saarland University, Saarbrücken, Germany

**Keywords:** microRNA, gene expression, transposition of the great arteries, heart failure, systemic left and right ventricle

## Abstract

**Background:**

Patients with transposition of the great arteries (TGA) have different connected systemic chambers and this determines the long-term morbidities and survival. Limited findings have been reported to systematically identify miRNA and mRNA expression levels in such cohorts of patients. In this study, we aimed to characterize miRNAs, mRNAs, and miRNA–mRNA interaction networks in patients with TGA, with a systemic left (LV) and right ventricle (RV).

**Materials and methods:**

Large panel of human miRNA and mRNA microarrays were conducted to determine the genome-wide expression profiles in the blood of 16 TGA-RV patients, 16 TGA-LV patients, and 16 age and gender-matched controls. Using real-time quantitative PCR (RT-qPCR), the differential expression level of a single miRNA was validated. Enrichment analyses of altered miRNA and mRNA expression levels were identified using bioinformatics tools.

**Results:**

Altered miRNA and mRNA expression levels were observed between TGA-RV and TGA-LV patients, together or separated, compared to controls. Among the deregulated miRNAs and mRNAs, 39 and 101 miRNAs were identified as significantly differentially expressed in patients with TGA (both TGA-RV and TGA-LV) and TGA-RV, when compared to matched controls. Furthermore, 51 miRNAs were identified as significantly differentially expressed in patients with TGA-RV when compared to patients with TGA-LV. RT-qPCR relative expression level was highly consistent with microarray analysis results. Similarly, 36 and 164 mRNAs were identified as significantly differentially expressed in patients with TGA (both TGA-RV and TGA-LV) and TGA-RV, when compared to matched controls. Additionally, miR-140-3p showed a higher expression level in patients with overt heart failure (FC = 1.54; *P* = 0.001) and miR-502-3p showed a higher expression level in patients died due to cardiac death (FC = 1.41; *P* = 0.011). Integrative analysis resulted in 21 and 23 target genes with higher and lower expression levels, respectively (*r* ≥ 0.50 and *P* < 0.05). These target genes (i.e., 21 and 23 target genes) showed an inverse direction of regulation with miRNA and exhibited a miRNA binding site position within the 3′UTR of the target gene.

**Conclusion:**

Our findings provide new insights into a potential molecular biomarker(s) for patients with TGA that may guide better risk stratification and the development of novel targeting therapies. Future studies are needed to investigate the potential significance of miRNAs and mRNAs in TGA-related cardiovascular diseases.

## Introduction

Congenital heart defects (CHDs) are the most common organ malformations affecting approximately 0.8–1.2% of live births worldwide (1). Despite a large amount of research carried out to define the etiological causes of CHDs, only ∼15% of cases can be attributable to a known genetic cause (1, 2). Congenital heart and vessel defects have heterogeneous forms of morphological and hemodynamic manifestations, including single or a combination of different anatomical malformations. Of these CHDs, transposition of the great arteries (TGA), is characterized by either ventriculo-arterial discordance alone (D-TGA), or a combination of both discordant connections, including atrio-ventricular and ventriculo-arterial discordance (L-TGA). Clinical manifestations of the two forms of TGAs range from severe postnatal cyanosis requiring immediate therapy and intervention at birth, as in the case of D-TGA. While children with D-TGA have a systemically left ventricle after arterial switch operation, they have a mostly better outcome, and fewer cardiac events and heart failure, older patients with D-TGA underwent the initial era of cardiac surgery only switch of circulation at atrial levels (atrial switch operation) and still have RV as a systemic chamber. Patients with L-TGA have congenital atrio-ventricular and ventriculo-arterial discordant connections and their blood pulmonary and systemic circulation and are considered physiologically, rather than anatomically, corrected. These patients still have the RV in a systemic position and have hemodynamic and cardiac outcomes similar to the patients with D-TGA after atrial switch operations. Both cohorts of patients (i.e., D-TGA after atrial switch operation and L-TGA) may have similar hemodynamic situations (RV in systemic position) but they still have genetically different determined congenital malformations. Different surgical operations aim to correct the hemodynamic and morphological situation in order to achieve physiological blood flow in pulmonary and systemic circulations. These surgical operations underwent several modifications in the last six decades. Patients with TGA, who still have RV in a systemic position are known to be at risk for heart failure (HF), atrial and ventricular arrhythmia, and sudden death (3, 4). The occurrence of overt HF has negative implications on the prognosis of CHD patients (5). At the beginning of the surgical era, patients with D-TGA who underwent atrial switch had significantly more failed systemic RV and severe cardiovascular events, and less survival than those patients with D-TGA after anatomical arterial switch repair. As a result, prevention or early treatment of HF is crucial to avoid the worsening of HF that is associated with a poor prognosis in these patients. Although the underlying genetic mechanisms of TGA development are poorly understood, several protein-coding genes are known to play a restricted functional role during cardiac development and function. However, the cellular and biological functions of most of these genes are still unclear. Gene expression during cardiac development is highly dynamic and strictly regulated post-transcriptionally via several regulators including microRNAs (miRNAs) (6, 7).

MiRNAs are non-coding, single-stranded RNAs (approximately 18–25 nucleotides in length) that generally bind to the 3’ untranslated regions (3’UTRs) of messenger RNAs (mRNAs) to suppress protein translation or cause mRNA degradation (8). Currently, there are 2300 “real and mature” miRNAs identified (9) which regulate almost every cellular and biological process, including processes regulating CHDs (10–15). Concerning patients with TGA, two studies published shortly afterward reported the miRNA expression levels in patients with TGA using either array-based reverse transcription and quantitative real-time PCR (RT-qPCR) (16) or single RT-qPCR analysis (17). In the first study, 11 miRNAs showed higher expression levels in patients with TGA after atrial switch operation of which miR-18a and miR-486-5p were negatively related to the contractility of the systemic right ventricle (16), whereas in the second study, miR-423-5p failed to be associated with the systolic function of the systemic right ventricle (17). Most recently, miR-183-3p was found to be an independent biomarker of worsening HF and thus may be used as an additional biomarker in the risk assessment of patients with TGA and systemic RV (10). These studies are indicating the important role of miRNA in CHDs and that an alteration in miRNA expression levels is associated with cardiovascular diseases and/or TGA-related manifestations. In patients with TGA (either D-TGA or L-TGA), however, integrative analysis of miRNA and mRNA expression levels is still lacking. Thus, it is conceivable that, in addition to an entire miRNA and mRNA profiling, the search for an inverse function for a miRNA whereby it can increase the translation of a specific target may demonstrate another layer of the molecular diversity of TGA and may potentially be a useful diagnostic and prognostic tool for therapy. Therefore, in this study, we identified the alteration in miRNA and mRNA expression levels in patients with D-TGA and a systemic RV or LV, as well as in patients with L-TGA, having already systemic RV. The results were compared to age and gender-matched healthy volunteers (HVs). We furthermore performed an integrated analysis to identify the mRNA targets of the deregulated miRNAs and to identify the potential miRNA(s) and/or mRNA (s) that might be involved in the mechanisms underlying TGA.

## Materials and methods

### Study population and sample collection

In total, 48 participants with TGA and healthy controls were prospectively recruited during the routine cardiac follow-up at the Department of Cardiology of Saarland University Hospital. Samples were collected from patients with TGA (*n* = 32) and age/gender-matched healthy controls (HVs, *n* = 16) (Mean ± SD: 20.09 ± 8.07 and 20.63 ± 8.69, respectively). Of the 32 patients with TGA enrolled in the study, 16 had a systemic morphological right ventricle (TGA-RV) and 16 patients had a systemic morphological left ventricle (TGA-LV) after arterial switch operation. All patients were regularly seen in our clinic on a 1-year basis and underwent the same study protocol that has been described in detail previously (12) including 12-lead electrocardiogram, transthoracic two-dimensional echocardiography, and laboratory tests (Table 1). A physical examination and two-dimensional echocardiography were carried out in all HVs to assess cardiac findings and status, including measurements of blood pressure, transcutaneous oxygen saturation, and two-dimensional echocardiography. The control group was mainly recruited at our institution and consisted of medical staff as well as medical students; however, to avoid selection bias, individuals from other, non-medical institutions were also enrolled. Blood samples (2.5 ml) were collected in PAXgene™ blood tubes (BD Biosciences, San Jose, CA, USA) and were stored at room temperature for 2 h to lysis blood cells before they were stored at −20°C until RNA, including miRNA isolation. The study complies with the declaration of Helsinki and good clinical practice guidelines. It was approved by the ethical board of the Saarland Medical Association. All patients and healthy controls gave written informed consent before enrollment into the study.

**TABLE 1:** Characteristics of enrolled controls and TGA patients.

Variables	TGA-RV (*n* = 16)		TGA-LV (*n* = 16)		Control (*n* = 16)		
	Median	IRQ	Median	IRQ	Median	IRQ	*P*-value
Age at follow-up (years)	18.5 (15–26.5)	11.5	20 (13.75–26.25)	12.5	19.5 (12.75–26.25)	13.5	0.9563*
Male sex	11/16 (68.75%)		11/16 (68.75%)		11/16 (68.75%)		
Patients with overt HF	2/16 (12.50%)		2/16 (12.50)				
Death	2/16(12.50%)		2/16 (12.50%)				
Transcutaneous oxygen saturation (%)	93 (91–95.25)	4.25	93.5 (92–96)	4	98 (97.75–99)	1.25	0.0001*
Systolic blood pressure (mmHg)	118.5 (106.5–126.5)	20	118 (115.75–135.25)	19.5	119 (113.5–126.5)	13	0.5941*
Diastolic blood pressure (mmHg)	64.5 (60.5–70.75)	10.25	63.5 (56.75–77.25)	20.5	67.5 (60.75–72)	11.25	0.8963*
NYHA functional class	1.5 (1–2)	1	1 (1–1)	0			0.1323**
NT-proBNP (pg/ml)	93.2 (51.75–265.425)	213.675	168.45 (104.725–407.45)	302.725			0.1964**
High sensitive troponin T (pg/ml)	3.5 (3–5.25)	2.25	4 (3–13.25)	10.25			0.4207**
Gamma GT (U/l)	67 (49.5–90)	40.5	69 (41.25–134.5)	93.25			0.9935**
Albumin (g/l)	48 (44.75–49.25)	4.5	46 (44–49)	5			0.5678**
eGFR (ml/min)	118.05 (84.725–131.9)	47.175	98.85 (85.325–116.675)	31.35			0.1567**
End-systolic volume of RV (ml)	77 (65.25–86.5)	21.25	52.5 (34.25–77.5)	43.25	42 (32–47.5)	15.5	0.0022*
End-diastolic volume of RV (ml)	159.5 (131.5–201.75)	70.25	123.5 (81.75–)	89.75	102.5 (84.25–128.5)	44.25	0.0079*
Ejection fraction of RV (%)	54.5 (49.75–60)	10.25	56 (54.75–58.75)	4	62 (58.75–63.25)	4.5	0.0028*
VTI above aortic valve (cm)	23.75 (22.15–26.075)	3.925	23.05 (21.5–26.275)	4.775	25.9 (24.2–28.525)	4.32	0.1186*

NYHA, New York Heart Association; HF, heart failure; VTI, velocity-time integral; eGFR, estimated glomerular filtration rate. Mean ± standard deviation was used. TGA-RV and TGA-LV compared to controls. *One-way non-parametric ANOVA (Kruskal–Wallis test). **Non-parametric Mann-Whitney U test.

### Purification and quality assessment

Total RNA including miRNAs was purified from blood samples using the PAXgene™ Blood miRNA Kit on the QIAcube™ robot (Qiagen, Hilden, Germany) following the Qiagen’s instructions, and DNase I treatment step was included for each sample (Qiagen). Only samples with A260/A280 ratio between 1.8 and 2.1 using NanoDrop 2000 Spectrophotometer (ThermoFisher Scientific, Waltham, MA, USA) and RNA Integrity Number (RIN) > 7 using Agilent 2100 Bioanalyzer (Agilent Technologies, Santa Clara, CA, USA) were included in the analysis.

### Analysis of miRNAs by microarray

The miRNA expression profiles in the blood samples of TGA-RV patients (*n* = 16) and HVs controls (*n* = 16) were obtained from our previously generated and published raw data using the SurePrint™ 8 × 60K Human miRNA platform (Agilent Technologies) (10). As for TGA-LV patients, miRNA profiling analysis was performed using the purified RNA fraction from 16 TGA-LV samples using the SurePrint™ 8 × 60K Human miRNA platform (Agilent Technologies). These platforms contain probes for the detection of 2,549 human miRNAs following Agilent Technologies’ instructions. Briefly, 125 ng RNA was labeled, hybridized to the miRNA microarray chip, washed, and the images were acquired using an Agilent DNA microarray scanner (Agilent Technologies) as previously described (18, 19). Generated data were imported into *R* statistical environment software (version *R*-4.1.2) for further statistical analysis.

### Analysis of mRNAs by microarray

Similarly, the expression level of mRNAs was performed using the same samples that were used for the miRNA microarray analysis by hybridization onto SurePrint G3 Human Gene Expression v3 8 × 60K microarrays, containing 50,599 biological features (Agilent Technologies), according to the manufacturer’s instructions with slight modification. mRNA expression profiling was carried out on TGA-RV (*n* = 16), TGA-LV (*n* = 16), and controls (*n* = 16). Briefly, 125 ng total RNA was reverse transcribed, amplified, labeled with cyanine-3 (Cy3), and subsequently hybridized to the mRNA microarray chip. Arrays were washed, and images were acquired using an Agilent DNA microarray scanner (Agilent Technologies). Finally, generated data were imported into R statistical environment software (version R-4.1.2) for further statistical analysis.

### Reverse Transcription and Quantitative Real-Time PCR (RT-qPCR) of miRNA

A group of differentially identified miRNAs from the microarray analysis were selected for further validation by RT-qPCR in the same samples that were used in the microarray analysis (n = 48). A total of 38 miRNAs were selected from the TGA (TGA-RV and TGA-LV)/controls, TGA-RV/TGA-LV, and TGA-RV/controls comparisons (Supplementary Table 1). These differentially expressed and identified miRNA were selected based on their significant abundance levels (adjusted *P*-value), fold change, and biological relevance in CHDs or other types of confounding cardiac and extracardiac abnormalities. The procedure has been complete as previously described (20, 21). Briefly, 75 ng total RNA was reverse transcribed to complementary DNA (cDNA) using the TaqMan™ MicroRNA Reverse Transcription Kit (ThermoFisher Scientific), according to ThermoFisher Scientific’s instructions. Following reverse transcription, 2.5 μl of the generated cDNA was pre-amplified using TaqMan™ PreAmp Master Mix (ThermoFisher Scientific). Finally, the abundance level of miRNAs was detected by RT-qPCR using Biomark HD/96.96 Dynamic Array™ IFC arrays (Fluidigm Corporation) as indicated in Fluidigm’s protocol for miRNA analysis (PN 68000130 E1). The generated data were extracted from the Biomark Data Collection software and were then further analyzed using the Fluidigm Real-Time PCR Analysis software (Fluidigm Corporation). Resulted cycle threshold (Ct) values were plotted individually for each miRNA, and only samples with Ct ≤ 35 were considered for analysis. Besides, No -Template Control (NTC) and RT-negative control were included in each run.

### Bioinformatics and statistical analysis

Using *R* software, features obtained from the raw data were filtered to exclude mRNAs and miRNAs, respectively with a detection rate of less than 50% in each group (i.e., patients and controls). Resulted expression signals were then quantile-normalized, log2-transformed and the differentially abundant miRNAs in the patients’ group compared to HVs samples were determined after applying an unpaired two-tailed *t*-test. *P*-values were corrected using the false discovery rate (FDR)-controlling procedure by Benjamini–Hochberg. Adjusted *P*-values smaller than 0.05 were considered significant and the volcano plot and heatmap were calculated based on log2. The fold-changes and adjusted *P*-values, Volcano plots of differentially abundant miRNAs and mRNAs were plotted using either GraphPad Prism (version 9.0.2) and/or R software (version *R*-4.1.2). To generate all plots shown, either base R functionality was used and/or functions from the ggplot2 v3.2.1, pheatmap v1.0.12, rcolorbrewer v1.1.2, igraph v1.2.6, viridis v0.5.1, and grepel v0.8.1. The packages data.tablev1.12.8, openxlsx v4.1.4, scales v1.1.0, stringr v1.4.0, and rfast v1.9.5 were used to implement common data manipulation tasks. Analysis of potential mRNA–miRNA interactions was performed using R with data.table v1.12.0, corrplot v0.84, and viridisLite v0.3.0. The predictive ability of each miRNA was evaluated using the receiver operating characteristic (ROC curve) and area under the curve (AUC). Adjusted *P*-values for each AUC value was calculated based on the *F*-test on logistic regression models trained with the corresponding variables, with a Benjamini Hochberg Multiple Comparison Test (FDR < 0.05). The whole miRNA and mRNA microarray datasets including the differentially expressed miRNAs and mRNAs are provided in the public repository Gene Expression Omnibus with the assigned accession number GSE179105. As for the RT-qPCR data, the relative quantitative procedure was used to detect the expression changes of single miRNAs and an unpaired two-tailed *t*-test. *P*-values were used to calculate the difference in significance levels. The small nuclear RNA (snRNA) RNU6B was chosen as a reference endogenous control for normalization as previously indicated for this type of sample (10–14, 18, 19, 22–24). The Spearman correlation coefficient was calculated for each significantly differently expressed miRNA-mRNA pair for the TGA groups (TGA-LV and TGA-RV) and controls, as well as *P*-values for this correlation. TargetScan 7.2^1^ (25), was used to predict the miRNA that exhibited a binding site within the 3′UTR region of the target genes, and the biological significance of significantly and negatively correlated miRNAs was assessed *in silico* with miEAA 2.0 (26).

## Results

### Characteristics of the study population

Forty-Eight participants (including 32 patients with TGA and 16 controls) were recruited for this study. The detailed clinical characteristics of participants are listed in Table 1. Controls and patients (TGA-RV and TGA-LV) were significantly different in terms of transcutaneous oxygen saturation (%) (*P* = 0.0001), end-systolic volume of RV (ml) (*P* = 0.0022), end-diastolic volume of RV (ml) (*P* = 0.0079), and ejection fraction of RV (%) (*P* = 0.0028). However, other clinical parameters, such as systolic and diastolic blood pressures and NYHA class and laboratory tests such as high-sensitive troponin T, Gamma GT, Albumin, and eGFR were not significantly different. Patients with TGA and RV have significantly larger end-systolic and end-diastolic volumes, than patients with TGA with LV, as a result of chronic pressure and volume overload of the morphologically RV (Table 1). Interestingly, the values of the biochemical marker NT-ProBNP were not significantly different between the TGA patients with right and left ventricles.

### Quality check and clustering analysis of miRNA and mRNA data

Unsupervised hierarchical clustering analyses of miRNAs, mRNAs, and samples, i.e., patients with TGA-RV and TGA-LV vs. controls based on average linkage and Euclidian distance of the significantly expressed miRNAs and mRNAs, were carried out. Forty-eight samples [TGA-RV (*n* = 16), TGA-LV (*n* = 16), and controls (*n* = 16)] were included in this study. Out of 48 samples, 46 [TGA-RV (*n* = 16), TGA-LV (*n* = 15), and controls (*n* = 15)] passed the miRNA microarray quality check and their expression levels were included in the further analysis, and two samples including [TGA-LV (*n* = 1) and control (*n* = 1)] were not passed the quality check and were excluded from further analysis. Similarly, 48 samples [TGA-RV (*n* = 16), TGA-LV (*n* = 16), and controls (*n* = 16)] passed the mRNA microarray quality check and their expression levels were included in the further analysis. As for the miRNA clustering analysis, patients with TGA-RV and TGA-LV, together, or separated, were clustered *versus* the controls. For this task, we included only the miRNAs with the highest expression variances (i.e., adjusted *P*-value and higher fold change). Although there was a highly significant and altered miRNA profile between patients with TGA vs. controls, TGA-RV vs. controls, and TGA-RV vs. TGA-LV, there was no clear clustering of samples based on any of the expressed miRNAs. There were clusters containing lower expression levels detected mostly in controls and/or in TGA either TGA-RV or TGA-LV, and the expression levels ranged from lower to higher in the TGA patients compared to controls. A more detailed distinction between the tested groups based on the unsupervised clustering dendrogram of miRNAs was, however, not conclusive (Supplementary Figures 1–3).

As for the mRNA clustering analysis, patients with TGA-RV and TGA-LV, together, or separated, were clustered versus the controls. The difference in mRNA expression in TGA-RV patients and controls revealed by unsupervised hierarchical clustering analysis of the mRNA exhibited an adjusted *P*-value of <0.05 and ≥1.5-fold change on both sides (i.e., lower, and higher expression levels). The sample dendrogram generated by hierarchical cluster analysis showed two major clusters, a cluster containing mostly TGA-RV patients and a second cluster containing most of the controls, indicating a specific mRNA expression pattern in the TGA-RV vs. the normal controls (Supplementary Figures 4, 5). Based on those two clusters, some mRNAs showed clearly differential expression levels in TGA-RV and/or expressed at a low level in the controls, and vice-versa. A clearer distinction between patients with both TGA-RV and TGA-LV and controls based on the hierarchical clustering of differentially expressed mRNA was, however, not possible.

### Screening of differentially expressed miRNAs and mRNA using microarray

**FIGURE 1:**
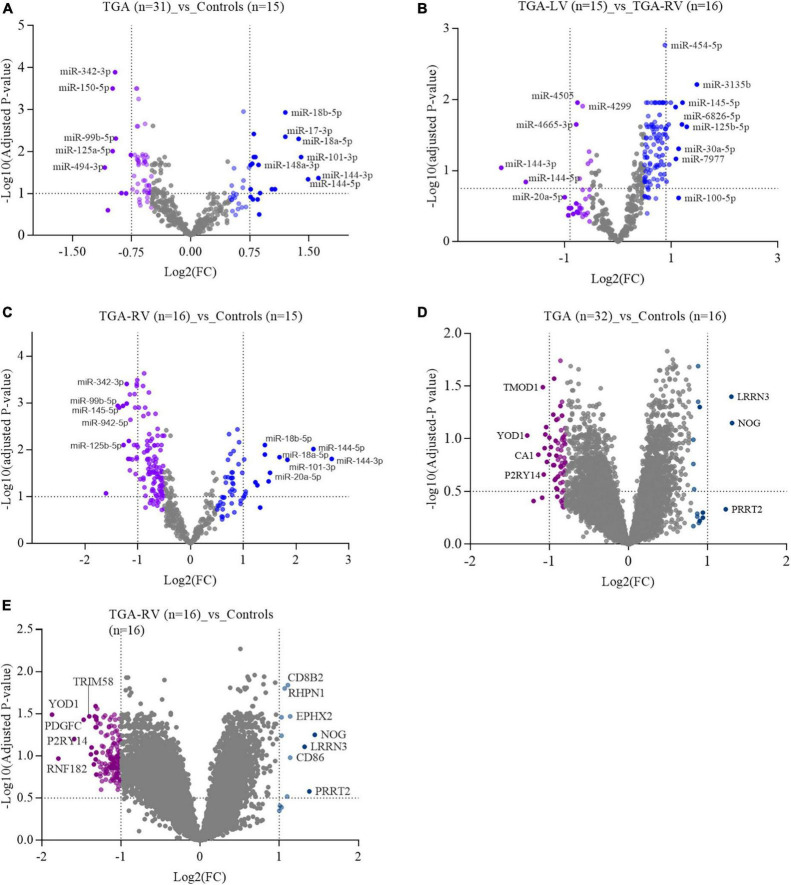
A volcano plot showing the log2 fold change of most significantly expressed miRNAs and miRNAs in blood samples of patients with TGA (TGA-RV and TGA-LV) compared to matched controls, as determined by microarray analysis.

Using the high-throughput SurePrint microarray platforms, the differential expression analyses were performed to identify miRNAs that showed either higher expression levels or lower expression levels in the blood samples collected from patients with TGA (TGA-RV and TGA-LV) compared to age and gender-matched controls. Considering only miRNAs exhibited an adjusted *P*-value of <0.05 and ≥1.5-fold change on both sides (i.e., lower, and higher expression levels), 39 and 101 miRNAs were identified as significantly differentially expressed in patients with TGA (both TGA-RV and TGA-LV) and TGA-RV when compared to matched controls (Table 2). Furthermore, 51 miRNAs were identified as significantly differentially expressed in patients with TGA-RV when compared to patients with TGA-LV (Table 2). However, no miRNAs were identified as significantly differentially expressed in patients with TGA-LV when compared to matched controls. The most differentially expressed lower and higher expressed miRNAs were visualized in a volcano plot (Figure 1). The levels of miRNAs differed between patients and controls are illustrated in the heatmap (Supplementary Figures 1–3).

**TABLE 2:** Significantly abundant miRNAs in the blood of TGA patients compared to controls as determined by microarray.

microRNA	Fold change	Adjusted *P*-value	Regulation
**(A)** **TGA patients (*n* = 31) vs. controls (*n* = 15)**			
miR-494-3p	0.47	0.0240	Lower
miR-150-5p	0.50	0.0003	Lower
miR-125a-5p	0.50	0.0099	Lower
miR-342-3p	0.51	0.0001	Lower
miR-99b-5p	0.52	0.0049	Lower
miR-145-5p	0.59	0.0120	Lower
miR-199a-5p	0.61	0.0384	Lower
miR-1246	0.61	0.0120	Lower
miR-93-3p	0.62	0.0003	Lower
miR-193b-3p	0.62	0.0025	Lower
miR-361-5p	0.62	0.0003	Lower
miR-15b-3p	0.63	0.0153	Lower
miR-6794-3p	0.63	0.0135	Lower
miR-532-3p	0.63	0.0025	Lower
miR-23a-3p	0.63	0.0161	Lower
miR-6125	0.63	0.0192	Lower
miR-4732-5p	0.63	0.0207	Lower
miR-942-5p	0.63	0.0172	Lower
miR-128-3p	0.63	0.0006	Lower
miR-23b-3p	0.65	0.0129	Lower
miR-629-3p	0.66	0.0117	Lower
miR-1275	0.66	0.0492	Lower
miR-139-5p	0.66	0.0192	Lower
miR-454-5p	0.67	0.0157	Lower
miR-6819-3p	1.59	0.0011	Higher
miR-103a-3p	1.59	0.0492	Higher
miR-29b-3p	1.67	0.0240	Higher
miR-590-5p	1.70	0.0208	Higher
miR-140-5p	1.71	0.0194	Higher
miR-106b-5p	1.74	0.0038	Higher
miR-29c-3p	1.74	0.0135	Higher
miR-301a-3p	1.77	0.0135	Higher
miR-148a-3p	1.81	0.0210	Higher
miR-17-3p	2.29	0.0044	Higher
miR-18b-5p	2.30	0.0012	Higher
miR-18a-5p	2.58	0.0051	Higher
miR-101-3p	2.64	0.0135	Higher
miR-144-5p	2.81	0.0455	Higher
miR-144-3p	3.08	0.0429	Higher
**(B) TGA-LV patients (*n* = 15) vs. TGA-RV**			
miR-4665-3p	0.58	0.0223	Lower
miR-4505	0.59	0.0110	Lower
miR-4299	0.63	0.0123	Lower
miR-1306-5p	1.50	0.0240	Higher
miR-21-3p	1.53	0.0346	Higher
miR-6803-3p	1.54	0.0224	Higher
miR-22-5p	1.55	0.0110	Higher
miR-378a-3p	1.56	0.0224	Higher
miR-574-3p	1.57	0.0240	Higher
miR-296-5p	1.57	0.0257	Higher
miR-1255b-5p	1.59	0.0240	Higher
miR-505-3p	1.59	0.0284	Higher
miR-501-3p	1.60	0.0427	Higher
miR-5690	1.60	0.0308	Higher
miR-3200-3p	1.61	0.0110	Higher
miR-193b-3p	1.62	0.0110	Higher
miR-93-3p	1.62	0.0206	Higher
miR-212-3p	1.63	0.0350	Higher
miR-4449	1.65	0.0110	Higher
miR-664a-5p	1.66	0.0240	Higher
miR-211-3p	1.66	0.0341	Higher
miR-378g	1.66	0.0259	Higher
miR-6073	1.68	0.0384	Higher
miR-629-3p	1.70	0.0110	Higher
miR-15b-3p	1.71	0.0308	Higher
miR-3200-5p	1.71	0.0492	Higher
miR-500b-5p	1.72	0.0164	Higher
miR-6794-3p	1.73	0.0110	Higher
miR-664a-3p	1.74	0.0350	Higher
miR-181b-5p	1.75	0.0240	Higher
miR-1260b	1.77	0.0405	Higher
miR-339-3p	1.79	0.0110	Higher
miR-3907	1.79	0.0110	Higher
miR-99a-5p	1.80	0.0110	Higher
miR-4732-5p	1.80	0.0308	Higher
miR-454-5p	1.84	0.0017	Higher
miR-99b-5p	1.85	0.0259	Higher
miR-340-5p	1.85	0.0259	Higher
miR-500a-5p	1.86	0.0308	Higher
miR-1246	1.87	0.0110	Higher
miR-339-5p	1.88	0.0240	Higher
miR-378a-5p	1.88	0.0484	Higher
miR-186-5p	1.89	0.0341	Higher
miR-378d	1.93	0.0223	Higher
miR-942-5p	1.99	0.0110	Higher
miR-6085	2.12	0.0127	Higher
miR-30a-5p	2.20	0.0492	Higher
miR-6826-5p	2.30	0.0223	Higher
miR-145-5p	2.31	0.0110	Higher
miR-125b-5p	2.44	0.0240	Higher
miR-3135b	2.79	0.0061	Higher
**(C) TGA-RV patients (*n* = 16) vs. controls (*n* = 15)**			
miR-99b-5p	0.39	0.0012	Lower
miR-145-5p	0.39	0.0013	Lower
miR-125a-5p	0.41	0.0012	Lower
miR-125b-5p	0.41	0.0079	Lower
miR-150-5p	0.43	0.0010	Lower
miR-342-3p	0.43	0.0004	Lower
miR-494-3p	0.44	0.0156	Lower
miR-3135b	0.44	0.0065	Lower
miR-484	0.45	0.0156	Lower
miR-1246	0.45	0.0007	Lower
miR-4428	0.45	0.0275	Lower
miR-942-5p	0.45	0.0023	Lower
miR-8485	0.47	0.0160	Lower
miR-4732-5p	0.48	0.0079	Lower
miR-6794-3p	0.48	0.0006	Lower
miR-15b-3p	0.48	0.0013	Lower
miR-409-3p	0.48	0.0329	Lower
miR-326	0.49	0.0145	Lower
miR-378a-5p	0.49	0.0156	Lower
miR-93-3p	0.49	0.0004	Lower
miR-339-5p	0.49	0.0079	Lower
miR-193b-3p	0.49	0.0004	Lower
miR-454-5p	0.50	0.0003	Lower
miR-199a-5p	0.50	0.0087	Lower
miR-629-3p	0.51	0.0013	Lower
miR-186-5p	0.52	0.0423	Lower
miR-574-3p	0.54	0.0004	Lower
miR-532-3p	0.54	0.0019	Lower
miR-5189-3p	0.54	0.0393	Lower
miR-6789-5p	0.54	0.0431	Lower
miR-361-5p	0.54	0.0002	Lower
miR-3200-5p	0.54	0.0114	Lower
miR-99a-5p	0.55	0.0013	Lower
miR-361-3p	0.57	0.0164	Lower
miR-191-5p	0.57	0.0106	Lower
miR-139-5p	0.57	0.0079	Lower
miR-505-3p	0.57	0.0065	Lower
miR-6125	0.57	0.0102	Lower
miR-151a-3p	0.57	0.0156	Lower
miR-664a-3p	0.57	0.0256	Lower
miR-30c-5p	0.57	0.0329	Lower
miR-3200-3p	0.58	0.0017	Lower
miR-128-3p	0.58	0.0006	Lower
miR-30b-5p	0.58	0.0300	Lower
miR-5690	0.58	0.0046	Lower
miR-4672	0.58	0.0195	Lower
miR-423-3p	0.58	0.0156	Lower
miR-23a-3p	0.58	0.0093	Lower
miR-574-5p	0.59	0.0300	Lower
miR-4732-3p	0.59	0.0329	Lower
miR-2861	0.59	0.0232	Lower
miR-92a-3p	0.59	0.0065	Lower
miR-7-1-3p	0.60	0.0058	Lower
miR-1275	0.61	0.0304	Lower
miR-4449	0.61	0.0170	Lower
miR-501-3p	0.61	0.0279	Lower
miR-5001-5p	0.62	0.0221	Lower
miR-7704	0.62	0.0303	Lower
miR-10a-5p	0.63	0.0080	Lower
miR-4721	0.63	0.0410	Lower
miR-1271-5p	0.63	0.0140	Lower
miR-1268b	0.63	0.0489	Lower
miR-6803-3p	0.63	0.0080	Lower
miR-23b-3p	0.63	0.0184	Lower
miR-6513-3p	0.63	0.0017	Lower
miR-212-3p	0.64	0.0233	Lower
miR-654-3p	0.64	0.0156	Lower
miR-296-5p	0.64	0.0284	Lower
miR-3180-3p	0.64	0.0327	Lower
miR-222-3p	0.64	0.0376	Lower
miR-3605-3p	0.65	0.0050	Lower
miR-324-3p	0.65	0.0036	Lower
miR-130b-5p	0.65	0.0079	Lower
miR-1268a	0.66	0.0326	Lower
miR-423-5p	0.66	0.0384	Lower
miR-6511b-3p	0.66	0.0238	Lower
miR-29b-3p	1.58	0.0384	Higher
miR-4713-3p	1.64	0.0384	Higher
miR-4665-3p	1.64	0.0164	Higher
miR-4433a-5p	1.66	0.0046	Higher
miR-29c-3p	1.70	0.0122	Higher
miR-3198	1.70	0.0405	Higher
miR-5581-5p	1.72	0.0304	Higher
miR-6131	1.72	0.0393	Higher
miR-1305	1.74	0.0304	Higher
miR-6740-5p	1.75	0.0398	Higher
miR-33b-3p	1.77	0.0106	Higher
miR-6819-3p	1.86	0.0037	Higher
miR-6717-5p	1.88	0.0203	Higher
miR-106b-5p	1.90	0.0069	Higher
miR-301a-3p	1.97	0.0310	Higher
miR-19a-3p	2.03	0.0160	Higher
miR-17-5p	2.34	0.0491	Higher
miR-18b-5p	2.65	0.0079	Higher
miR-17-3p	2.67	0.0126	Higher
miR-96-5p	2.78	0.0471	Higher
miR-20a-5p	2.86	0.0310	Higher
miR-18a-5p	3.22	0.0144	Higher
miR-101-3p	3.59	0.0164	Higher
miR-144-5p	5.01	0.0097	Higher
miR-144-3p	6.40	0.0156	Higher

An unpaired two-tailed t-test was used to calculate the P-value. TGA-RV, transposition of the great arteries with a systemic right ventricle; TGA-LV, transposition of the great arteries with a systemic left ventricle; AUC, area under the curve.

Similarly, the high-throughput SurePrint microarray platforms were used to identify mRNAs that showed either higher expression levels or lower expression levels in the blood samples collected from patients with TGA (TGA-RV and TGA-LV) compared to age and gender-matched controls. Considering only mRNAs exhibited an adjusted *P*-value of <0.05 and ≥1.5-fold change on both sides (i.e., lower, and higher expression levels), 36 and 164 mRNA were identified as significantly differentially expressed in patients with TGA (both TGA-RV and TGA-LV) and TGA-RV when compared to matched controls (Table 3). However, no mRNAs were identified as significantly differentially expressed in patients with TGA-LV when compared to matched TGA-RV and controls. The most differentially expressed lower and higher mRNAs are visualized in a volcano plot (Figure 1). The levels of miRNAs differed between patients and controls are illustrated in the heatmap (Supplementary Figures 4, 5).

**TABLE 3:** Significantly abundant mRNAs in the blood of TGA patients compared to controls as determined by microarray.

(A) TGA patients (*n* = 32) vs. controls (*n* = 16)							
mRNA	Fold change	Adjusted *P*-value	Regulation	mRNA	Fold change	Adjusted *P*-value	Regulation
TMOD1	0.47	0.0321	Lower	LRRN3	2.47	0.0394	Higher
PRSS33	0.52	0.0270	Lower	TRABD2A	1.84	0.0448	Higher
SFRP2	0.55	0.0493	Lower	CD8B2	1.84	0.0206	Higher
RGS1	0.55	0.0485	Lower	PLEKHB1	1.71	0.0210	Higher
SRXN1	0.55	0.0181	Lower	IQCN	1.71	0.0340	Higher
RHCE	0.56	0.0444	Lower	CD248	1.70	0.0306	Higher
TFDP1	0.59	0.0298	Lower	IL7R	1.69	0.0449	Higher
DNAJC6	0.59	0.0476	Lower	TMIGD2	1.66	0.0274	Higher
HRH4	0.59	0.0433	Lower	C1QTNF6	1.62	0.0480	Higher
PTPRF	0.60	0.0286	Lower	NRCAM	1.61	0.0357	Higher
NRP1	0.60	0.0466	Lower	ASIC1	1.56	0.0449	Higher
RIPOR3	0.60	0.0204	Lower	NPM3	1.56	0.0382	Higher
DACH1	0.61	0.0444	Lower	GSDMB	1.56	0.0425	Higher
STOM	0.61	0.0347	Lower	NOSIP	1.54	0.0362	Higher
TMC5	0.62	0.0350	Lower	PTPRCAP	1.54	0.0237	Higher
MPP1	0.64	0.0440	Lower	PBX4	1.52	0.0203	Higher
OXER1	0.64	0.0300	Lower	OFD1	1.50	0.0209	Higher
CLEC5A	0.65	0.0218	Lower	SCML4	1.50	0.0278	Higher
**(B) TGA-RV patients (*n* = 16) vs. controls (*n* = 16)**							
**mRNA**	**Fold change**	**Adjusted *P*-value**	**Regulation**	**mRNA**	**Fold change**	**Adjusted *P*-value**	**Regulation**
YOD1	0.27	0.0321	Lower	EPHX2	2.21	0.0336	Higher
PDGFC	0.36	0.0373	Lower	CD8B2	2.15	0.0146	Higher
TRIM58	0.38	0.0340	Lower	RHPN1	2.10	0.0157	Higher
TENT5C	0.40	0.0343	Lower	KLK1	2.04	0.0349	Higher
XK	0.40	0.0456	Lower	PLEKHB1	1.95	0.0326	Higher
TMOD1	0.40	0.0256	Lower	TCF7	1.95	0.0419	Higher
JAZF1	0.40	0.0455	Lower	CD248	1.93	0.0135	Higher
RNF11	0.40	0.0346	Lower	FHIT	1.91	0.0420	Higher
SNCA	0.40	0.0370	Lower	CD8B	1.90	0.0457	Higher
FOXO3	0.41	0.0275	Lower	CCR10	1.81	0.0451	Higher
GCLC	0.41	0.0403	Lower	C12orf57	1.81	0.0411	Higher
KAT2B	0.42	0.0414	Lower	NPM3	1.78	0.0180	Higher
FURIN	0.43	0.0348	Lower	PCSK4	1.76	0.0114	Higher
TPM1	0.44	0.0330	Lower	C1QTNF6	1.75	0.0437	Higher
RHOBTB1	0.45	0.0460	Lower	IQCN	1.74	0.0365	Higher
SLC7A5	0.45	0.0450	Lower	ASIC1	1.74	0.0141	Higher
PIP5K1B	0.45	0.0368	Lower	FXYD2	1.73	0.0401	Higher
CTNNAL1	0.46	0.0345	Lower	TMIGD2	1.72	0.0370	Higher
RAP2A	0.46	0.0439	Lower	NRCAM	1.72	0.0317	Higher
CALD1	0.47	0.0451	Lower	HIST1H1D	1.71	0.0375	Higher
FNBP1L	0.47	0.0321	Lower	NOSIP	1.69	0.0326	Higher
ARHGAP6	0.48	0.0371	Lower	TLE2	1.66	0.0356	Higher
CREG1	0.49	0.0362	Lower	AMIGO1	1.66	0.0459	Higher
TBCEL	0.50	0.0445	Lower	SYNE4	1.66	0.0349	Higher
GCNT1	0.50	0.0320	Lower	PTPRCAP	1.64	0.0340	Higher
GSPT1	0.50	0.0401	Lower	SELENOM	1.64	0.0458	Higher
RIOK3	0.50	0.0445	Lower	SOX8	1.64	0.0457	Higher
PDCD10	0.50	0.0374	Lower	KLHL34	1.63	0.0209	Higher
ABCB10	0.50	0.0458	Lower	NPY4R	1.62	0.0404	Higher
TTC7B	0.51	0.0326	Lower	GSDMB	1.61	0.0444	Higher
DNAJC6	0.51	0.0340	Lower	LIG1	1.61	0.0110	Higher
ADIPOR1	0.51	0.0346	Lower	FAM174B	1.60	0.0350	Higher
RGS1	0.52	0.0449	Lower	SLC27A5	1.60	0.0430	Higher
NRP1	0.52	0.0358	Lower	RPL36	1.60	0.0255	Higher
TMC5	0.52	0.0167	Lower	HPDL	1.58	0.0460	Higher
BNIP3L	0.52	0.0322	Lower	SCML4	1.58	0.0255	Higher
RIPOR3	0.52	0.0118	Lower	CD7	1.57	0.0353	Higher
STOM	0.53	0.0341	Lower	COL6A1	1.57	0.0373	Higher
DNM3	0.53	0.0349	Lower	VILL	1.56	0.0456	Higher
NIPSNAP3A	0.53	0.0432	Lower	MYL6B	1.56	0.0454	Higher
F13A1	0.53	0.0422	Lower	MTFP1	1.56	0.0330	Higher
FAM104A	0.53	0.0313	Lower	C19orf48	1.55	0.0369	Higher
TFDP1	0.53	0.0372	Lower	GPC2	1.54	0.0311	Higher
XYLT1	0.53	0.0451	Lower	SFI1	1.54	0.0328	Higher
CLIC2	0.53	0.0117	Lower	MRNIP	1.54	0.0124	Higher
RHCE	0.53	0.0420	Lower	CD27	1.54	0.0406	Higher
MKRN1	0.53	0.0373	Lower	MST1R	1.53	0.0404	Higher
TUBB1	0.54	0.0463	Lower	ZFP90	1.53	0.0441	Higher
PTPRF	0.54	0.0192	Lower	TEDC1	1.53	0.0341	Higher
SRXN1	0.54	0.0158	Lower	ATP5IF1	1.53	0.0314	Higher
DAB2	0.54	0.0461	Lower	SPEG	1.52	0.0440	Higher
SFRP2	0.54	0.0453	Lower	ZNF692	1.52	0.0120	Higher
SLC25A37	0.54	0.0433	Lower	STMN3	1.52	0.0452	Higher
PIP4K2A	0.55	0.0179	Lower	ZNF444	1.52	0.0115	Higher
GP6	0.56	0.0413	Lower	TMEM160	1.51	0.0358	Higher
GUCY1B1	0.56	0.0473	Lower	TESPA1	1.51	0.0314	Higher
HRH4	0.56	0.0422	Lower	ADAMTS13	1.51	0.0156	Higher
EPB41	0.57	0.0372	Lower	PBX4	1.51	0.0458	Higher
VEGFC	0.57	0.0438	Lower	ATAD3B	1.50	0.0431	Higher
HEXIM1	0.57	0.0385	Lower				
HNMT	0.57	0.0444	Lower				
DACH1	0.58	0.0357	Lower				
GAPT	0.58	0.0363	Lower				
RPIA	0.58	0.0412	Lower				
MPP1	0.58	0.0333	Lower				
COPS2	0.58	0.0458	Lower				
CHMP4B	0.59	0.0323	Lower				
P2RY12	0.59	0.0460	Lower				
FAM117A	0.59	0.0458	Lower				
BMP6	0.59	0.0370	Lower				
DCAF6	0.59	0.0242	Lower				
MPL	0.60	0.0446	Lower				
CA8	0.60	0.0410	Lower				
GOLM1	0.60	0.0473	Lower				
ACPP	0.60	0.0317	Lower				
STON1	0.60	0.0458	Lower				
RNF10	0.60	0.0373	Lower				
BHLHE40	0.61	0.0370	Lower				
SNX3	0.61	0.0411	Lower				
SPTA1	0.61	0.0348	Lower				
WNK1	0.61	0.0421	Lower				
CLEC5A	0.61	0.0403	Lower				
RAB27B	0.61	0.0442	Lower				
KLRB1	0.61	0.0459	Lower				
ODC1	0.62	0.0345	Lower				
OPTN	0.62	0.0453	Lower				
ATP6V0C	0.62	0.0358	Lower				
TAL1	0.62	0.0460	Lower				
DHX29	0.62	0.0460	Lower				
ZNF469	0.62	0.0345	Lower				
UBE2E3	0.63	0.0459	Lower				
VCL	0.63	0.0359	Lower				
HK1	0.63	0.0366	Lower				
KIF1B	0.63	0.0482	Lower				
LRP12	0.63	0.0440	Lower				
PCDH9	0.64	0.0464	Lower				
TFPI	0.64	0.0459	Lower				
LTBP1	0.64	0.0342	Lower				
VLDLR	0.64	0.0451	Lower				
BMP2K	0.65	0.0375	Lower				
RNF14	0.65	0.0418	Lower				
SELENOK	0.65	0.0334	Lower				
C2orf76	0.66	0.0310	Lower				
SLC6A4	0.66	0.0456	Lower				
CTNNA1	0.66	0.0461	Lower				

An unpaired two-tailed *t*-test was used to calculate the *P*-value. TGA-RV, transposition of the great arteries with a systemic right ventricle.

### Confirmation by single real-time RT-qPCR

**FIGURE 2:**
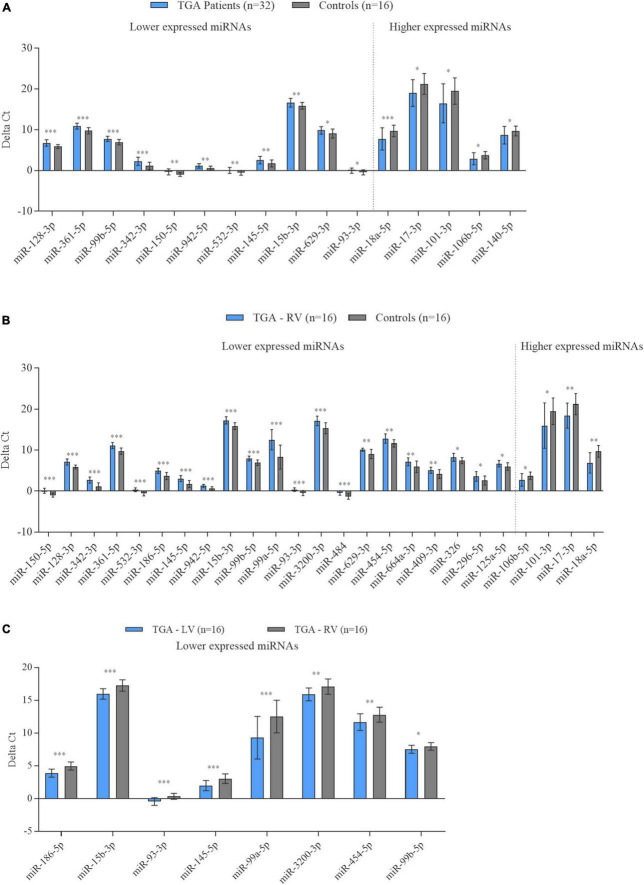
Expression levels of miRNAs in the blood samples of patients with TGA (TGA-RV and TGA-LV) compared to controls determined by RT-qPCR. Data are presented as the mean ΔCt of either TGA-RV or TGA-LV and both (TGA) and matched controls (lower ΔCt, higher abundance level). RNU RNU6B was used as an endogenous control for the normalization of miRNAs, respectively. Unpaired-two-tailed t-test and mean ± SD was used to evaluate differences in expression level. *P* < 0.05 with FDR adjustment was considered statistically significant (*P < 0.05; **P < 0.01; ***P < 0.001).

To further validate the microarray results, a total of 38 miRNAs were selected to determine their differential expression levels by RT-qPCR in the TGA (TGA-RV and TGA-LV)/controls, TGA-RV/TGA-LV and TGA-RV/controls comparisons as shown in Supplementary Table 2. These miRNAs were chosen based on their differential expression level in each patient with either TGA-RV, TGA-LV, or both and matched controls. The results of RT-qPCR analysis showed that the expression patterns of most of these miRNAs were consistent with the microarray results (Figure 2). Compared with matched controls, 16 out of 25 miRNAs showed a significant same direction of expression changes as the microarray results in TGA, including 11 miRNAs with lower expression levels in TGA (both TGA-RV and TGA-LV) and 5 miRNAs with higher expression levels in TGA (Figure 2). The rest miRNAs (9 out 25 miRNAs tested by RT-qPCR) namely miR-125a-5p, miR-18b-5p, miR-454-5p, miR-29c-3p, miR-590-5p, miR-1275, miR-6794-3p, miR-193b-3p, and miR-29b-3p showed a same direction of expression changes (i.e., higher, and lower expression changes) with no statistical significance changes. As for the comparison between patients with only TGA-RV and matched controls, 25 tested miRNAs by RT-qPCR showed a significant same direction of expression changes as the microarray results, including 21 miRNAs with lower expression levels and 4 miRNAs with higher expression levels (Figure 2). The rest miRNAs (9 out 34 miRNAs tested by RT-qPCR) showed the same direction of expression changes (i.e., higher, and lower expression changes) with no statistically significant changes for 7 miRNAs including 5 miRNAs with lower expression levels (miR-1275, miR-339-5p, miR-574-3p, miR-29c-3p, miR-19a-3p, miR-6794, and miR-193b-3p) and 2 miRNAs with higher expression level (miR-18b-5p and miR-29c-3p) in patients with TGA-RV as compared to controls. Additionally, two miRNAs namely miR-29c-3p and miR-29b-3p showed an inverse direction of regulation, i.e., a lower mRNA expression level by RT-qPCR analysis and higher expression levels by microarray analysis. As for the compassion within TGA patients, 8 miRNAs namely miR-186-5p, miR-15b-3p, miR-93-3p, miR-145-5p, miR-99a-5p, miR-3200-3p, miR-454-5p, and miR-99b-5p showed a significant same direction of expression changes as the microarray results. All these miRNAs showed lower expression levels in patients with TGA-RV compared to patients with TGA-LV (Figure 2).

### Integrative analysis identified miRNA–mRNA interaction networks for TGA

**FIGURE 3:**
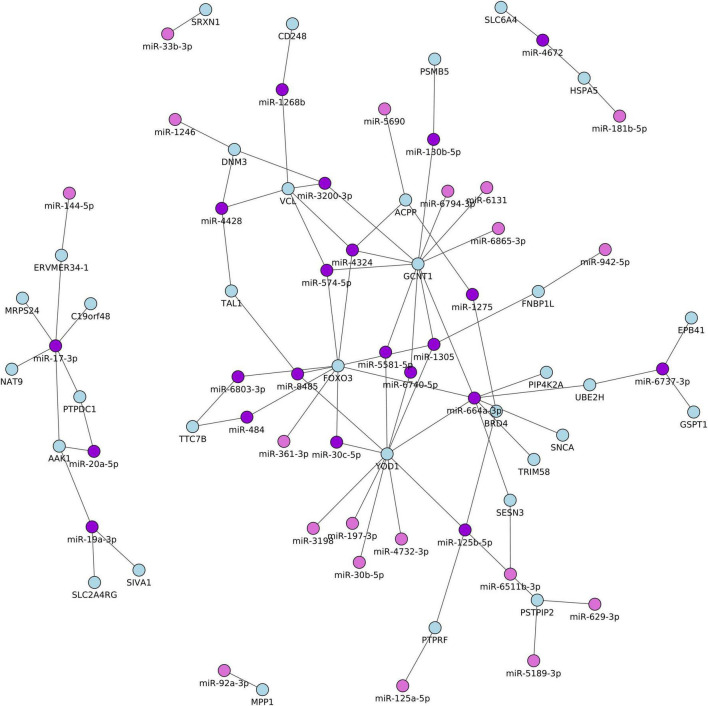
Network of the differentially expressed miRNA and their potential target mRNAs with inverse correlations between TGA patients and controls. Only pairs with a correlation coefficient of ≥–0.5, *P* < 0.05, and a miRNA binding site within the 3’UTR of the target gene.

To gain insights into the broader biological context in which the differentially identified miRNAs and mRNAs operate in patients with TGA and controls, we performed an integrative network analysis using significantly differentially expressed miRNAs and the mRNAs which were identified in Table 2 and Table 3. In addition, we computed the correlation coefficients (r) of miRNA–mRNA pairs which are differentially expressed between patients with RV-TGA compared to controls. We only considered pairs with a correlation coefficient of ≥−0.5 and a miRNA binding site within the 3’UTR of the target gene. A total of 83 miRNA-mRNA correlations were identified, including 28 with lower miRNA and higher mRNA expression levels and 55 with higher miRNA and lower mRNA expression levels (Supplementary Table 3). As shown in Figure 3, the integrative analysis resulted in 21 higher expressed mRNAs and 12 lower expressed miRNAs and 23 lower expressed mRNAs, and 28 higher expressed miRNAs. These target mRNAs (i.e., 21 and 23 target mRNAs) showed an inverse direction of regulation with miRNA (i.e., 12 and 28 miRNAs, 40 miRNAs) and exhibited a miRNA binding site position within the 3’UTR of the target gene. Interestingly, out of 40 miRNAs, 27 miRNAs were identified to be expressed in myocardium tissue, as indicated in Supplementary Table 4. Additionally, results provided by overrepresentation analyses for the negatively correlated miRNAs indicated that some miRNAs are related to cardiovascular-related manifestations including cardiac arrhythmias, cardiomegaly, heart failure, myocardial infarction, and myocarditis. Also showed terms typically associated with CHDs related pathways (Supplementary Table 4).

### miRNA–mRNA is associated with the occurrence of overt heart failure and death

In order to further identify important miRNA and mRNA that are associated with OHF occurrence and might lead to death, the differential expression analysis was carried out. As for the miRNA analysis, one miRNA namely miR-140-3p showed a higher expression level in patients with overt heart failure (FC = 1.54; *P* = 0.001) and one miRNA namely miR-502-3p showed a higher expression level in patients who died due to sudden cardiac death (FC = 1.41; *P* = 0.011). While considering only mRNAs which exhibited an adjusted *P*-value of <0.05 and ≥1.5-fold change on both sides (i.e., lower, and higher expression levels), 124 differentially expressed mRNAs were identified in patients with OHF compared with subjects without OHF, including 75 genes with lower expression levels and 47 genes with higher expression levels in OHF patients compared to patients without OHF (Supplementary Table 5). Similarly, 69 differentially expressed mRNAs were identified in blood samples collected from patients who died due to sudden cardiac death compared with surviving subjects, including 41 genes with lower expression levels and 28 genes with higher expression levels (Supplementary Table 6). Given the availability of both higher expressed miRNAs (i.e., miR-140-3p and miR-502-3p) and lower expressed mRNAs (i.e., 75 and 41 mRNAs with lower expression levels in OHF patients and patients who died due to sudden cardiac death, respectively), the integrative analysis was carried out to identify the inverse direction of regulation i.e., a lower mRNA expression level and an elevated miRNA expression level. Using the miRWalk algorithm, 11,292 and 14,754 potential target genes for the miR-140-3p and miR-502-3p were predicted, respectively. These potential target genes were cross-matched against the 75 and 41 genes identified by microarray analysis with lower expression levels in patients with OHF and patients that died due to sudden cardiac death. As a result, 42 and 38 target genes were yielded. These yielded target genes (i.e., 42 and 38 target genes) exhibited a miRNA binding site position within the 3’UTR of the target genes and were further investigated for a functional role in congenital heart diseases including in patients with TGA.

### Diagnostic value of the combination of miRNAs and heart failure marker

To explore the diagnostic value of significantly identified miRNAs, we analyzed their diagnostic value in combination with heart failure markers including NT-ProBNP, high-sensitivity troponin T, and eGFR (27) (Supplementary Table 7). As shown in Table 4, the AUC value of each heart failure marker was 0.68, 0.62, and 0.59 for NT-ProBNP, high-sensitivity troponin T, and eGFR, respectively. The combination between the heart failure markers with the expression level of miRNAs has increased the power of diagnosis of AUC value to > 0.9 for some miRNAs (Supplementary Table 7), indicating that these miRNAs can be used along with heart failure markers to distinguish patients with TGA-RV from patients with TGA-LV.

**TABLE 4:** Diagnostic power of miRNA in combination with heart failure markers in TGA-RV patients compared to TGA-LV patients.

NT-ProBNP + miRNA	AUC	Adjusted *P*-value	High-sensitivity troponin T + miRNA	AUC	Adjusted *P*-value	eGFR + miRNA	AUC	Adjusted *P*-value
NT-ProBNP	0.68	0.230	high-sensitivity troponin T	0.62	0.143	eGFR	0.59	0.165
miR-6794-3p	0.93	0.002	miR-6794-3p	1.00	0.002	miR-1246	0.96	0.008
miR-574-3p	0.93	0.014	miR-3135b	0.98	0.002	miR-3135b	0.92	0.006
miR-3135b	0.92	0.002	miR-1246	0.98	0.003	miR-574-3p	0.91	0.012
miR-1246	0.91	0.006	miR-4449	0.96	0.008	miR-6794-3p	0.89	0.006
miR-4449	0.91	0.008	miR-22-5p	0.93	0.008	miR-454-5p	0.87	0.002
miR-4324	0.89	0.010	miR-574-3p	0.93	0.012	miR-4449	0.87	0.009
miR-652-3p	0.89	0.006	miR-1306-5p	0.92	0.013	miR-1306-5p	0.87	0.030
miR-1306-5p	0.89	0.017	miR-193b-3p	0.91	0.006	miR-145-5p	0.84	0.010
miR-6803-3p	0.88	0.014	miR-652-3p	0.90	0.008	miR-4433a-5p	0.83	0.041
miR-454-5p	0.87	0.002	miR-6766-3p	0.90	0.034	miR-22-5p	0.83	0.008

A receiver operating characteristic (ROC) curve analysis was performed, and the area under the ROC curve (AUC) was calculated to evaluate the diagnostic value. Adjusted *P*-values calculated based on the *F*-test on logistic regression models trained with the corresponding variables, with a Benjamini Hochberg Multiple Comparison Test (FDR = 0.05).

## Discussion

In this study, we reported the miRNA and mRNA expression patterns in patients with TGA (TGA-RV and TGA-LV) and healthy controls. With miRNA and mRNA profiling along with RT-qPCR validation, we found a group of differentially expressed miRNAs and mRNAs in patients with TGA compared to controls. Integrative analysis identified 83 miRNA-mRNA correlations between miRNA (40 miRNAs) and mRNAs (33 target mRNA). In each identified mRNA, a miRNA binding site position within the 3’UTR of the target mRNA gene was observed. Interestingly, out of correlated 40 miRNAs, 27 miRNAs were identified to be expressed in myocardium tissue, as indicated in Supplementary Table 4. Overrepresentation enrichment analysis of all mRNAs regulated by miRNAs (a miRNA binding site position within the 3’UTR) highlighted diseases and pathways that play a role in cardiovascular-related manifestations including heart failure, myocardial infarction, and myocarditis.

TGA, where the aorta arising from the RV and pulmonary artery from the LV is the main morphological manifestation in patients with both cohorts of D-TGA and L-TGA. While blood circulation, and not the anatomy in L-TGA is congenitally corrected by additional discordance between both atria and ventricles (congenitally corrected TGA/double discordance), patients with D-TGA have separated pulmonary and systemic circulation and are presented with severe cyanosis without mixing possibilities by enlargement of patent foramen ovale (PFO) (Rashkind maneuver) and keeping the patent ductus arteriosus (PDA) open. In the last decades (60–80) many patients with D-TGA underwent a palliative operative procedure to correct the blood circulation at atrial levels by keeping the RV as a systemic chamber. However, in the last 3 decades’ anatomical correction through switching the great arteries to the anatomically and physiologically related ventricles has become the standard method for the treatment of d-TGA. The RV in the systemic position is mostly a pressure and volume overload. Several myocardial remodeling may occur including ventricle enlargement and the development of myocardial fibrosis resulting in low cardiac output and less physical performance. In addition, RV dilatation and myocardial fibrosis are high-risk factors for ventricular arrhythmia and sudden cardiac death (28). Patients with D-TGA after arterial switch operations have LV in a systemic position may develop less life-threatening cardiac events such as ventricular arrhythmia and myocardial dysfunction as is the case in patients with systemic RV. Long-term cardiac morbidities are mainly present from aortic dilatation, and supravalvular aortic stenosis (SVAS) (29). In contrast to other results. In our cohort, patients with morphologically systemic RV have significantly enlarged end-systolic and end-diastolic chamber diameters as expected, due to the chronic pressure and volume overload of the systemic RV (30–32). Interestingly, the conventional biomarkers for heart failure NT proBNP and other biochemical markers for the liver, which indicate precordial congestion, are similar and not significantly different in both groups. This may indicate compensated hemodynamic conditions and cardiac output in patients with systemic RV. The ejection fraction of the systemic chamber in both groups was not significantly different (Table 1). In addition, the flow velocity integral over the aortic valve was similar in both groups, indicating similar cardiac output. This may explain the similar values of NT-ProBNP in both groups indicating similar compensated systemic chamber performance. Our results concerning the abundance of miRNA, however, show significantly different expression patterns, which may indicate the difference in genetic background between both entities, and the differences in hemodynamic and morphologic conditions. The RV in a systemic position undergoes different remodeling in size, morphology, and function. As we found the systolic and diastolic parameters of RV in a systemic position were significantly enlarged. A significant increase in fibrosis is detectable in the myocardium of the RV with chronic pressure and volume overload. Whether miRNA indicates such remodeling is still unclear.

In this study, we identified six miRNAs including miR-125a-5p, miR-125b-5p, miR-181b-5p, miR-19a-3p, miR-20a-5p, and miR-30b-5p were significantly associated with heart failure (HF) (Supplementary Table 4). Of these six miRNAs, the miR-125 family was previously reported to be dysregulated and significantly correlated with HF and many other cardiac manifestations (12, 33–35). Also, miR-19, miR-181, miR-125, and miR-30 levels were associated with myocardium levels during the development of diabetic cardiomyopathy and failing hearts (33, 36), and the miR-30 and miR-19 families were frequently reported to be dysregulated in patients with the acute coronary syndrome (ACS) (37). Consistent with other studies (34, 36), miR-140 was dysregulated in our study in TGA patients with HF compared to TGA patients without HF, suggesting that miR-140 may play an important role in the pathogenesis of TGA. Additionally, miR-502 was dysregulated in patients with chronic congestive heart failure (CHF) (38) and reported as a biomarker in coronary artery disease (CAD) (37). Additionally, miR-20a expression was strongly correlated with echocardiographic RV functional parameters in patients with chronic thromboembolic pulmonary hypertension (CTEPH), and miR-20a along with miR-17 has a potential value in the diagnosis of CTEPH (39). MiR-664a-3p was expressed in the blood collected in patients with cardioembolic stroke compared to controls (40). This miRNA (miR-664a) exhibited a binding site within the 3’UTR of many correlated target genes as shown in Figure 3, including FOXO3, GCNT1, PIP4K2A, SESN3, SNCA, TRIM58, UBE2H, and YOD1. Of these genes, FOXO3 increased in expression in systolic HF patients compared with controls (41), and SIVA1 along with EGR1 serve as targets to counteract apoptosis in cardiac tissue (42). Interestingly, of our detected mRNA targets, Heat Shock Protein A5 (HSPA5), which is a direct target of miR-181b-5p, contributes to starvation-induced autophagy and apoptosis in cardiomyocytes (43). EPB41 protein isoforms are differentially compartmentalized in the heart and form specific complexes with proteins central to cardiomyocyte Ca (2+) metabolism (44). Other genes, which have been correlated negatively with the miRNAs, have not yet been reported to be related to any biological function in cardiovascular diseases and/or TGA-related manifestations. The identification of integrative experimentally validated mRNA targets which have a binding site for the above-mentioned HF-related miRNAs may lead to a possible novel signature related to TGA, provide new prognostic parameters, and ultimately even generate targets for novel approaches to diagnosis and possible treatment. Intriguingly, many of the biological pathways identified (Supplementary Table 4) have been associated with the development of cardiovascular complications (45). Previous studies reported that miR-423-5p can be used as a strong prognostic biomarker for HF patients along with many other miRNAs including miR-423-3p, miR-21, miR-23, miR-18a, miR-18b, miR-106, miR-301a, and miR-128 (46). All these miRNAs showed an altered expression level in our studied subjects i.e., TGA patients compared to matched controls. Although Tutarel et al. (17) failed to find a correlation between the miR-423-5p and cardiopulmonary parameters in patients with a systemic right ventricle and reduced ejection fraction, we found a lower expression level of miR-423-5p and miR-423-3p in TGA-RV patients compared to matched controls. Moreover, D’Alessandra et al. found an association between NT-ProBNP concentration and miR-423-5p left ventricular end-diastolic volume (LVEDV) suggesting that miR-423-5p can be used as an independent predictor in patients with LVEDV (47). Additionally, the expression levels of miR-21, miR-126, and miR-423-5p were associated with the prognosis of acute decompensated HF (48). It is of note that miR-99b-5p, miR-145-5p, miR-125a-5p, miR-125b-5p, miR-150-5p, miR-23a-3p, miR-15b-3p, showed the most lower expression level in TGA-RV and TGA-LV patients compared to controls. Of these miRNAs, 150-5p exerts anti-apoptotic functions by directly suppressing distinct pro-apoptotic genes (49) or by inhibiting p53 activity which is a major inducer of apoptosis (50). Therefore, a lack or decreased level *of* miR-150-5p might activate apoptosis signaling in cardiomyocytes, which is crucial for the progression of heart failure. Moreover, a lower expression level of miR-150-5p was correlated with overt heart failure in patients with univentricular hearts (UVH) (12). Another miRNA, miR-23a-3p has cardioprotective effects and plays a crucial role in the induction of angiogenesis in the ischemic heart after myocardial infarction along with other miRNAs, including miR-19a, miR-21-5p, miR-22, miR-29, and miR-125b-5p that enhance cardiomyocyte survival, function, and attenuate cardiac fibrosis (51). In pathological response, it is legitimate to hypothesize that alteration in miRNA expression in HF displayed a pattern similar to that observed in many other cardiovascular diseases, and similar to that in our studied patients and lately may lead to TGA.

In the current study, the integrative miRNAs and mRNAs networking analysis were generated using blood samples collected from TGA patients and controls, not from cardiac tissues, which usually delivers a specific signature of which miRNA(s), mRNA(s), and/or protein(s) is expressed exclusively and/or ubiquitously in TGA patients and TGA related subgroups (i.e., TGA-RV and TGA-LV). Therefore, the identified signature may lead to discovering novel biomarkers to characterize the TGA rather than providing information on underlying functional mechanisms in TGA patients. The analysis of patients’ heart tissues would be also a highly interesting objective. However, the analysis of heart tissue does not help to corroborate our findings on finding a biomarker for the diagnosis and possible prognosis.

We would like to point out that this study has also a number of limitations. The small number of patients and controls enrolled is certainly a criticism. In particular, the number of patients with OHF and patients who died due to sudden cardiac death is too low to assess the prognostic and diagnostic values of miR-140-3p and miR-502-3p, respectively. Hence, a larger cohort of patients and a larger control group should be evaluated to provide further insights into the role of miRNAs and their target mRNAs in these patients. Moreover, in the present study, the discriminative or diagnostic power of the identified miRNAs in combination with the heart failure markers (NT-ProBNP, high-sensitivity troponin T, and eGFR) was carried out between the TGA-RV and TGA-LV, and lack of these clinical parameters (i.e., NT-ProBNP, high-sensitivity troponin T, and eGFR) in control group makes it impossible to discriminate between TGA and controls. However, the strength of the study is that it included a large number of miRNAs and mRNAs screened in TGA patients and controls in order to identify the integrative network between the miRNAs-mRNA that helps in characterizing TGA patients and controls. To our knowledge, no such data is currently available in the literature. In conclusion, based on our findings, we identified a set of deregulated miRNAs and mRNAs in TGA-RV and TGA-LV patients, separately or together, compared to controls. Since the identified miRNAs, mRNAs and their integrative network have also been reported to play a role in the context of several cardiovascular diseases and/or CHD-related manifestations, it is legitimate to speculate about a possible role of the identified miRNAs and mRNAs for TGA patients. The alteration of the expression levels of these identified miRNAs and mRNAs provides new candidates for further analysis, which may contribute to understanding the development of CHD in the future.

## Data availability statement

Publicly available datasets were analyzed in this study. This data can be found here: https://www.ncbi.nlm.nih.gov/geo/, GSE215941, GSE215940, and GSE215939.

## Ethics statement

The studies involving human participants were reviewed and approved by the Institutional Review Board approval/Ethikvotum Ärztekammer des Saarlandes: [Institutional Review Board (No. 07/18)]. Written informed consent to participate in this study was provided by the participants’ legal guardian/next of kin.

## Author contributions

EM and HA-K contributed equally to this work. MA-H performed experimental work and wrote the manuscript. MA-H, VW, and SR performed statistical and bioinformatics analyses. MA-H, EM, and HA-K designed the study, supervised the project, and edited the manuscript. TR-H and HA-K recruited and examined controls, diagnosed patients, and collected blood samples. All authors read and approved the final manuscript.

## Footnotes

1
www.targetscan.org

## Supplementary material

Supplementary Figure 1 Unsupervised hierarchical clustering (Euclidian distance, complete linkage) of the patients with TGA-RV and controls based on the differentially expressed miRNAs with the significant highest variance.

Supplementary Figure 2 Unsupervised hierarchical clustering (Euclidian distance, complete linkage) of the patients with TGA-RV and TGA-LV based on the differentially expressed miRNAs with the significant highest variance.

Supplementary Figure 3 Unsupervised hierarchical clustering (Euclidian distance, complete linkage) of the patients with TGA and controls based on the differentially expressed miRNAs with the significant highest variance.

Supplementary Figure 4 Unsupervised hierarchical clustering (Euclidian distance, complete linkage) of the patients with TGA and controls based on the differentially expressed mRNAs with the significant highest variance.

Supplementary Figure 5 Unsupervised hierarchical clustering (Euclidian distance, complete linkage) of the patients with TGA-RV and controls based on the differentially expressed mRNAs with the significant highest variance.
