# Supplementary material for: Expression profiling analysis reveals key microRNA–mRNA interactions in patients with transposition of the great arteries and systemic left and right ventricles
Source: Front Cardiovasc Med. 2023 Jan 12;9:1056427. doi: 10.3389/fcvm.2022.1056427 (PMC9878113; doi:10.3389/fcvm.2022.1056427)
Supplement: Supplementary file 1 [file Table_1.DOCX]

**Supplementary Tables**

**SUPPLEMENTARY TABLE 1 |** miRBase ID and assays ID of the miRNAs for validation by RT-qPCR.

| **miRBase ID** | **Assay ID** | **Strategy** |
| --- | --- | --- |
| hsa-miR-128-3p | Assay ID: 002216 | TaqMan® Assays and Arrays |
| hsa-miR-361-5p | Assay ID:000554 | TaqMan® Assays and Arrays |
| hsa-miR-99b-5p | Assay ID:000436 | TaqMan® Assays and Arrays |
| hsa-miR-342-3p | Assay ID:002260 | TaqMan® Assays and Arrays |
| hsa-miR-150-5p | Assay ID: 000473 | TaqMan® Assays and Arrays |
| hsa-miR-942-5p | Assay ID:002187 | TaqMan® Assays and Arrays |
| hsa-miR-532-3p | Assay ID:002355 | TaqMan® Assays and Arrays |
| hsa-miR-145-5p | Assay ID: 002278 | TaqMan® Assays and Arrays |
| hsa-miR-15b-3p | Assay ID:000390 | TaqMan® Assays and Arrays |
| hsa-miR-629-3p | Assay ID:001562 | TaqMan® Assays and Arrays |
| hsa-miR-93-3p | Assay ID:002139 | TaqMan® Assays and Arrays |
| hsa-miR-18a-5p | Assay ID:002422 | TaqMan® Assays and Arrays |
| hsa-miR-17-3p | Assay ID:002421 | TaqMan® Assays and Arrays |
| hsa-miR-101-3p | Assay ID:002253 | TaqMan® Assays and Arrays |
| hsa-miR-106b-5p | Assay ID:000442 | TaqMan® Assays and Arrays |
| hsa-miR-140-5p | Assay ID: 001187 | TaqMan® Assays and Arrays |
| hsa-miR-125a-5p | Assay ID:002198 | TaqMan® Assays and Arrays |
| hsa-miR-18b-5p | Assay ID:002217 | TaqMan® Assays and Arrays |
| hsa-miR-454-5p | Assay ID:001996 | TaqMan® Assays and Arrays |
| hsa-miR-29c-3p | Assay ID:000587 | TaqMan® Assays and Arrays |
| hsa-miR-590-5p | Assay ID:001984 | TaqMan® Assays and Arrays |
| hsa-miR-1275 | Assay ID:002840 | TaqMan® Assays and Arrays |
| hsa-miR-6794-3p | Assay ID:466341_mat | TaqMan® Assays and Arrays |
| hsa-miR-193b-3p | Assay ID:002467 | TaqMan® Assays and Arrays |
| hsa-miR-29b-3p | Assay ID: 000413 | TaqMan® Assays and Arrays |
| hsa-miR-186-5p | Assay ID: 002285 | TaqMan® Assays and Arrays |
| hsa-miR-99a-5p | Assay ID: 000435 | TaqMan® Assays and Arrays |
| hsa-miR-3200-3p | Assay ID:241643_mat | TaqMan® Assays and Arrays |
| hsa-miR-484 | Assay ID: 001821 | TaqMan® Assays and Arrays |
| hsa-miR-664a-3p | Assay ID: 002897 | TaqMan® Assays and Arrays |
| hsa-miR-409-3p | Assay ID:002332 | TaqMan® Assays and Arrays |
| hsa-miR-326 | Assay ID: 000542 | TaqMan® Assays and Arrays |
| hsa-miR-296-5p | Assay ID: 000527 | TaqMan® Assays and Arrays |
| hsa-miR-21-3p | Assay ID:002438 | TaqMan® Assays and Arrays |
| hsa-miR-500a-5p | Assay ID:002428 | TaqMan® Assays and Arrays |
| hsa-miR-339-5p | Assay ID:002257 | TaqMan® Assays and Arrays |
| hsa-miR-211-3p | Assay ID: 477502_mat | TaqMan® Assays and Arrays |
| hsa-miR-574-3p | Assay ID:002349 | TaqMan® Assays and Arrays |

**SUPPLEMENTARY TABLE 2 |** Validation of the differentially expressed miRNAs in the blood of patients with TGA compared controls as determined by RT-qPCR.

| **MicroRNA** | **miRBase ID** | **Assay ID** | **TGA Patients (mean)** | **Controls (mean)** | **Log Differnce (∆∆Ct)** | **Fold Change** | **P value** | **Regulation** |
| --- | --- | --- | --- | --- | --- | --- | --- | --- |
| miR-128-3p | hsa-miR-128-3p | Assay ID: 002216 | 6.74 | 5.88 | 0.86 | 0.55 | 0.00003 | Lower |
| miR-361-5p | hsa-miR-361-5p | Assay ID:000554 | 10.87 | 9.75 | 1.12 | 0.46 | 0.00003 | Lower |
| miR-99b-5p | hsa-miR-99b-5p | Assay ID:000436 | 7.75 | 6.96 | 0.80 | 0.58 | 0.00040 | Lower |
| miR-342-3p | hsa-miR-342-3p | Assay ID:002260 | 2.25 | 1.11 | 1.14 | 0.45 | 0.00043 | Lower |
| miR-150-5p | hsa-miR-150-5p | Assay ID: 000473 | -0.34 | -0.97 | 0.63 | 0.64 | 0.00098 | Lower |
| miR-942-5p | hsa-miR-942-5p | Assay ID:002187 | 1.14 | 0.60 | 0.54 | 0.69 | 0.00118 | Lower |
| miR-532-3p | hsa-miR-532-3p | Assay ID:002355 | 0.03 | -0.57 | 0.60 | 0.66 | 0.00377 | Lower |
| miR-145-5p | hsa-miR-145-5p | Assay ID: 002278 | 2.53 | 1.73 | 0.80 | 0.58 | 0.00517 | Lower |
| miR-15b-3p | hsa-miR-15b-3p | Assay ID:000390 | 16.62 | 15.86 | 0.76 | 0.59 | 0.00972 | Lower |
| miR-629-3p | hsa-miR-629-3p | Assay ID:001562 | 9.84 | 9.07 | 0.77 | 0.59 | 0.02535 | Lower |
| miR-93-3p | hsa-miR-93-3p | Assay ID:002139 | -0.03 | -0.47 | 0.44 | 0.74 | 0.03147 | Lower |
| miR-18a-5p | hsa-miR-18a-5p | Assay ID:002422 | 7.75 | 9.69 | -1.94 | 3.82 | 0.00224 | Higher |
| miR-17-3p | hsa-miR-17-3p | Assay ID:002421 | 18.99 | 21.22 | -2.23 | 4.69 | 0.01385 | Higher |
| miR-101-3p | hsa-miR-101-3p | Assay ID:002253 | 16.49 | 19.48 | -2.99 | 7.95 | 0.01423 | Higher |
| miR-106b-5p | hsa-miR-106b-5p | Assay ID:000442 | 2.89 | 3.75 | -0.85 | 1.80 | 0.01735 | Higher |
| miR-140-5p | hsa-miR-140-5p | Assay ID: 001187 | 8.67 | 9.69 | -1.02 | 2.03 | 0.04051 | Higher |
| miR-125a-5p | hsa-miR-125a-5p | Assay ID:002198 | 6.52 | 6.00 | 0.53 | 0.69 | 0.05381 | Lower |
| miR-18b-5p | hsa-miR-18b-5p | Assay ID:002217 | 10.35 | 12.00 | -1.65 | 3.14 | 0.06634 | Higher |
| miR-454-5p | hsa-miR-454-5p | Assay ID:001996 | 12.24 | 11.65 | 0.59 | 0.66 | 0.07435 | Lower |
| miR-29c-3p | hsa-miR-29c-3p | Assay ID:000587 | 8.91 | 9.37 | -0.46 | 1.38 | 0.20824 | Higher |
| miR-590-5p | hsa-miR-590-5p | Assay ID:001984 | 4.83 | 5.24 | -0.40 | 1.32 | 0.29579 | Higher |
| miR-1275 | hsa-miR-1275 | Assay ID:002840 | 7.15 | 6.77 | 0.38 | 0.77 | 0.34405 | Lower |
| miR-6794-3p | hsa-miR-6794-3p | Assay ID:466341_mat | 3.67 | 2.96 | 0.70 | 0.61 | 0.35116 | Lower |
| miR-193b-3p | hsa-miR-193b-3p | Assay ID:002467 | 1.29 | 1.08 | 0.20 | 0.87 | 0.61775 | Lower |
| miR-29b-3p | hsa-miR-29b-3p | Assay ID: 000413 | 13.66 | 13.58 | 0.08 | 0.94 | 0.87422 | Lower |
|  |  |  |  |  |  |  |  |  |
|  |  |  |  |  |  |  |  |  |
| **MicroRNA** | **miRBase ID** | **Assay ID** | **TGA-RV (mean)** | **Controls (mean)** | **Log Differnce (∆∆Ct)** | **Fold Change** | **P value** | **Regulation** |
| miR-150-5p | hsa-miR-150-5p | Assay ID: 000473 | 0.09 | -0.97 | 1.07 | 0.48 | 0.00001 | Lower |
| miR-128-3p | hsa-miR-128-3p | Assay ID: 002216 | 7.11 | 5.88 | 1.23 | 0.43 | 0.00001 | Lower |
| miR-342-3p | hsa-miR-342-3p | Assay ID:002260 | 2.70 | 1.11 | 1.59 | 0.33 | 0.00001 | Lower |
| miR-361-5p | hsa-miR-361-5p | Assay ID:000554 | 11.10 | 9.75 | 1.35 | 0.39 | 0.00002 | Lower |
| miR-532-3p | hsa-miR-532-3p | Assay ID:002355 | 0.36 | -0.57 | 0.93 | 0.53 | 0.00002 | Lower |
| miR-186-5p | hsa-miR-186-5p | Assay ID: 002285 | 4.98 | 3.68 | 1.30 | 0.41 | 0.00004 | Lower |
| miR-145-5p | hsa-miR-145-5p | Assay ID: 002278 | 3.05 | 1.73 | 1.32 | 0.40 | 0.00005 | Lower |
| miR-942-5p | hsa-miR-942-5p | Assay ID:002187 | 1.31 | 0.60 | 0.70 | 0.61 | 0.00005 | Lower |
| miR-15b-3p | hsa-miR-15b-3p | Assay ID:000390 | 17.26 | 15.86 | 1.40 | 0.38 | 0.00007 | Lower |
| miR-99b-5p | hsa-miR-99b-5p | Assay ID:000436 | 7.97 | 6.96 | 1.02 | 0.49 | 0.00007 | Lower |
| miR-99a-5p | hsa-miR-99a-5p | Assay ID: 000435 | 12.52 | 8.32 | 4.20 | 0.05 | 0.00014 | Lower |
| miR-93-3p | hsa-miR-93-3p | Assay ID:002139 | 0.37 | -0.47 | 0.83 | 0.56 | 0.00020 | Lower |
| miR-3200-3p | hsa-miR-3200-3p | Assay ID:241643_mat | 17.10 | 15.33 | 1.77 | 0.29 | 0.00040 | Lower |
| miR-484 | hsa-miR-484 | Assay ID: 001821 | -0.45 | -1.30 | 0.86 | 0.55 | 0.00042 | Lower |
| miR-629-3p | hsa-miR-629-3p | Assay ID:001562 | 10.12 | 9.07 | 1.05 | 0.48 | 0.00218 | Lower |
| miR-454-5p | hsa-miR-454-5p | Assay ID:001996 | 12.80 | 11.65 | 1.16 | 0.45 | 0.00332 | Lower |
| miR-664a-3p | hsa-miR-664a-3p | Assay ID: 002897 | 7.15 | 5.95 | 1.20 | 0.43 | 0.00855 | Lower |
| miR-409-3p | hsa-miR-409-3p | Assay ID:002332 | 5.09 | 4.20 | 0.90 | 0.54 | 0.01080 | Lower |
| miR-326 | hsa-miR-326 | Assay ID: 000542 | 8.25 | 7.43 | 0.81 | 0.57 | 0.01214 | Lower |
| miR-296-5p | hsa-miR-296-5p | Assay ID: 000527 | 3.58 | 2.59 | 0.99 | 0.50 | 0.01907 | Lower |
| miR-125a-5p | hsa-miR-125a-5p | Assay ID:002198 | 6.69 | 6.00 | 0.69 | 0.62 | 0.02829 | Lower |
| miR-106b-5p | hsa-miR-106b-5p | Assay ID:000442 | 2.75 | 3.75 | -1.00 | 2.00 | 0.03295 | Higher |
| miR-101-3p | hsa-miR-101-3p | Assay ID:002253 | 15.99 | 19.48 | -3.49 | 11.24 | 0.03967 | Higher |
| miR-17-3p | hsa-miR-17-3p | Assay ID:002421 | 18.41 | 21.22 | -2.81 | 7.03 | 0.00856 | Higher |
| miR-18a-5p | hsa-miR-18a-5p | Assay ID:002422 | 6.90 | 9.69 | -2.79 | 6.90 | 0.00070 | Higher |
|  |  |  |  |  |  |  |  |  |
|  |  |  |  |  |  |  |  |  |
| **MicroRNA** | **miRBase ID** | **Assay ID** | **TGA-LV (mean)** | **TGA-RV (mean)** | **Log Differnce (∆∆Ct)** | **Fold Change** | **P value** | **Regulation** |
| miR-186-5p | hsa-miR-186-5p | Assay ID: 002285 | 3.90 | 4.98 | -1.08 | 2.11 | 0.00003 | Higher |
| miR-15b-3p | hsa-miR-15b-3p | Assay ID:000390 | 15.98 | 17.26 | -1.28 | 2.43 | 0.00017 | Higher |
| miR-93-3p | hsa-miR-93-3p | Assay ID:002139 | -0.42 | 0.37 | -0.79 | 1.73 | 0.00024 | Higher |
| miR-145-5p | hsa-miR-145-5p | Assay ID: 002278 | 2.01 | 3.05 | -1.05 | 2.07 | 0.00048 | Higher |
| miR-99a-5p | hsa-miR-99a-5p | Assay ID: 000435 | 9.30 | 12.52 | -3.22 | 9.31 | 0.00392 | Higher |
| miR-3200-3p | hsa-miR-3200-3p | Assay ID:241643_mat | 15.91 | 17.10 | -1.19 | 2.28 | 0.00401 | Higher |
| miR-454-5p | hsa-miR-454-5p | Assay ID:001996 | 11.67 | 12.80 | -1.13 | 2.19 | 0.01267 | Higher |
| miR-99b-5p | hsa-miR-99b-5p | Assay ID:000436 | 7.53 | 7.97 | -0.44 | 1.36 | 0.04512 | Higher |
| miR-21-3p | hsa-miR-21-3p | Assay ID:002438 | 10.06 | 11.93 | -1.87 | 3.64 | 0.07658 | Higher |
| miR-500a-5p | hsa-miR-500a-5p | Assay ID:002428 | 4.49 | 2.28 | 2.21 | 0.22 | 0.08824 | Lower |
| miR-296-5p | hsa-miR-296-5p | Assay ID: 000527 | 2.78 | 3.58 | -0.80 | 1.74 | 0.09180 | Higher |
| miR-629-3p | hsa-miR-629-3p | Assay ID:001562 | 9.56 | 10.12 | -0.56 | 1.47 | 0.09408 | Higher |
| miR-942-5p | hsa-miR-942-5p | Assay ID:002187 | 0.98 | 1.31 | -0.32 | 1.25 | 0.10631 | Higher |
| miR-339-5p | hsa-miR-339-5p | Assay ID:002257 | 3.96 | 4.43 | -0.47 | 1.38 | 0.29891 | Higher |
| miR-6794-3p | hsa-miR-6794-3p | Assay ID:466341_mat | 4.12 | 3.22 | 0.90 | 0.54 | 0.30264 | Higher |
| miR-664a-3p | hsa-miR-664a-3p | Assay ID: 002897 | 6.59 | 7.15 | -0.56 | 1.47 | 0.33565 | Lower |
| miR-211-3p | hsa-miR-211-3p | Assay ID: 477502_mat | 15.18 | 15.52 | -0.35 | 1.27 | 0.43343 | Higher |
| miR-193b-3p | hsa-miR-193b-3p | Assay ID:002467 | 1.49 | 1.08 | 0.41 | 0.75 | 0.51712 | Higher |
| miR-574-3p | hsa-miR-574-3p | Assay ID:002349 | 4.45 | 4.25 | 0.20 | 0.87 | 0.72014 | Lower |

- An unpaired two-tailed t-test was used to calculate the *p*-value.

**SUPPLEMENTARY TABLE 3 |** Correlation between miRNA and mRNA expression levels (r ≥ 0.5 and P < 0.05)

| 1. **Lower miRNA and higher mRNA expression level** | | | | | |
| --- | --- | --- | --- | --- | --- |
| **miRNA** | **mRNA** | **Median miRNA** | **Median mRNA** | **r** | **P-value** |
| miR-1246 | DNM3 | 2.17 | 6.10 | -0.51 | 0.0413 |
| miR-125a-5p | PTPRF | 4.19 | 4.63 | -0.56 | 0.0244 |
| miR-125b-5p | BRD4 | 5.03 | 10.39 | -0.57 | 0.0205 |
| miR-125b-5p | PSTPIP2 | 5.03 | 6.15 | -0.52 | 0.0398 |
| miR-125b-5p | PTPRF | 5.03 | 4.63 | -0.75 | 0.0008 |
| miR-125b-5p | YOD1 | 5.03 | 8.06 | -0.56 | 0.0248 |
| miR-1268b | CD248 | 3.21 | 4.15 | -0.50 | 0.0471 |
| miR-1268b | VCL | 3.21 | 9.53 | -0.57 | 0.0210 |
| miR-1275 | ACPP | 2.60 | 6.35 | -0.58 | 0.0179 |
| miR-1275 | BRD4 | 2.60 | 10.39 | -0.58 | 0.0194 |
| miR-1305 | FNBP1L | 4.77 | 4.90 | -0.60 | 0.0146 |
| miR-1305 | FOXO3 | 4.77 | 10.28 | -0.73 | 0.0014 |
| miR-1305 | GCNT1 | 4.77 | 5.52 | -0.67 | 0.0045 |
| miR-1305 | YOD1 | 4.77 | 8.06 | -0.55 | 0.0263 |
| miR-130b-5p | GCNT1 | 1.92 | 5.52 | -0.54 | 0.0326 |
| miR-130b-5p | PSMB5 | 1.92 | 9.16 | -0.60 | 0.0146 |
| miR-144-5p | ERVMER34-1 | 6.99 | 5.38 | -0.55 | 0.0273 |
| miR-17-3p | AAK1 | 5.31 | 7.95 | -0.62 | 0.0099 |
| miR-17-3p | C19orf48 | 5.31 | 7.82 | -0.64 | 0.0081 |
| miR-17-3p | ERVMER34-1 | 5.31 | 5.38 | -0.60 | 0.0138 |
| miR-17-3p | MRPS24 | 5.31 | 11.36 | -0.62 | 0.0109 |
| miR-17-3p | NAT9 | 5.31 | 8.67 | -0.70 | 0.0025 |
| miR-17-3p | PTPDC1 | 5.31 | 5.48 | -0.54 | 0.0296 |
| miR-181b-5p | HSPA5 | 2.37 | 7.56 | -0.54 | 0.0292 |
| miR-197-3p | YOD1 | 6.36 | 8.06 | -0.53 | 0.0362 |
| miR-19a-3p | AAK1 | 7.47 | 7.95 | -0.56 | 0.0233 |
| miR-19a-3p | SIVA1 | 7.47 | 10.27 | -0.68 | 0.0037 |
| miR-19a-3p | SLC2A4RG | 7.47 | 10.99 | -0.72 | 0.0016 |
|  |  |  |  |  |  |
| 1. **Higher miRNA and lower mRNA expression level** | | | | | |
| **miRNA** | **mRNA** | **Median miRNA** | **Median mRNA** | **r** | **P-value** |
| miR-20a-5p | AAK1 | 9.39 | 7.95 | -0.57 | 0.0213 |
| miR-20a-5p | PTPDC1 | 9.39 | 5.48 | -0.56 | 0.0239 |
| miR-30b-5p | YOD1 | 10.29 | 8.06 | -0.53 | 0.0334 |
| miR-30c-5p | FOXO3 | 9.82 | 10.28 | -0.55 | 0.0289 |
| miR-30c-5p | YOD1 | 9.82 | 8.06 | -0.66 | 0.0056 |
| miR-3198 | YOD1 | 4.91 | 8.06 | -0.51 | 0.0432 |
| miR-3200-3p | DNM3 | 2.22 | 6.10 | -0.53 | 0.0350 |
| miR-3200-3p | GCNT1 | 2.22 | 5.52 | -0.51 | 0.0427 |
| miR-3200-3p | VCL | 2.22 | 9.53 | -0.51 | 0.0413 |
| miR-33b-3p | SRXN1 | 3.09 | 10.15 | -0.52 | 0.0400 |
| miR-361-3p | FOXO3 | 6.34 | 10.28 | -0.57 | 0.0220 |
| miR-4324 | ACPP | 1.68 | 6.35 | -0.80 | 0.0002 |
| miR-4324 | FOXO3 | 1.68 | 10.28 | -0.61 | 0.0129 |
| miR-4324 | GCNT1 | 1.68 | 5.52 | -0.61 | 0.0118 |
| miR-4324 | VCL | 1.68 | 9.53 | -0.83 | 0.0001 |
| miR-4428 | DNM3 | 1.83 | 6.10 | -0.69 | 0.0034 |
| miR-4428 | TAL1 | 1.83 | 5.43 | -0.51 | 0.0427 |
| miR-4428 | VCL | 1.83 | 9.53 | -0.58 | 0.0194 |
| miR-4672 | HSPA5 | 2.48 | 7.56 | -0.52 | 0.0387 |
| miR-4672 | SLC6A4 | 2.48 | 4.72 | -0.61 | 0.0113 |
| miR-4732-3p | YOD1 | 4.75 | 8.06 | -0.55 | 0.0263 |
| miR-484 | FOXO3 | 8.47 | 10.28 | -0.58 | 0.0182 |
| miR-484 | TTC7B | 8.47 | 7.21 | -0.59 | 0.0151 |
| miR-5189-3p | PSTPIP2 | 2.55 | 6.15 | -0.54 | 0.0297 |
| miR-5581-5p | GCNT1 | 4.24 | 5.52 | -0.68 | 0.0039 |
| miR-5581-5p | YOD1 | 4.24 | 8.06 | -0.63 | 0.0085 |
| miR-5690 | ACPP | 2.36 | 6.35 | -0.51 | 0.0441 |
| miR-574-5p | FOXO3 | 3.25 | 10.28 | -0.68 | 0.0040 |
| miR-574-5p | GCNT1 | 3.25 | 5.52 | -0.63 | 0.0091 |
| miR-574-5p | VCL | 3.25 | 9.53 | -0.53 | 0.0330 |
| miR-6131 | GCNT1 | 5.21 | 5.52 | -0.54 | 0.0297 |
| miR-629-3p | PSTPIP2 | 2.85 | 6.15 | -0.67 | 0.0045 |
| miR-6511b-3p | SESN3 | 2.46 | 6.75 | -0.51 | 0.0456 |
| miR-664a-3p | FOXO3 | 4.88 | 10.28 | -0.57 | 0.0202 |
| miR-664a-3p | GCNT1 | 4.88 | 5.52 | -0.56 | 0.0227 |
| miR-664a-3p | PIP4K2A | 4.88 | 10.71 | -0.67 | 0.0045 |
| miR-664a-3p | SESN3 | 4.88 | 6.75 | -0.64 | 0.0082 |
| miR-664a-3p | SNCA | 4.88 | 12.42 | -0.60 | 0.0140 |
| miR-664a-3p | TRIM58 | 4.88 | 12.19 | -0.61 | 0.0118 |
| miR-664a-3p | UBE2H | 4.88 | 9.59 | -0.59 | 0.0152 |
| miR-664a-3p | YOD1 | 4.88 | 8.06 | -0.76 | 0.0006 |
| miR-6737-3p | EPB41 | 3.56 | 6.39 | -0.65 | 0.0067 |
| miR-6737-3p | GSPT1 | 3.56 | 8.15 | -0.66 | 0.0055 |
| miR-6737-3p | UBE2H | 3.56 | 9.59 | -0.71 | 0.0022 |
| miR-6740-5p | GCNT1 | 5.67 | 5.52 | -0.56 | 0.0230 |
| miR-6740-5p | YOD1 | 5.67 | 8.06 | -0.51 | 0.0420 |
| miR-6794-3p | GCNT1 | 1.88 | 5.52 | -0.59 | 0.0165 |
| miR-6803-3p | FOXO3 | 2.29 | 10.28 | -0.52 | 0.0398 |
| miR-6803-3p | TTC7B | 2.29 | 7.21 | -0.51 | 0.0432 |
| miR-6865-3p | GCNT1 | 2.23 | 5.52 | -0.55 | 0.0283 |
| miR-8485 | FOXO3 | 2.59 | 10.28 | -0.51 | 0.0432 |
| miR-8485 | TAL1 | 2.59 | 5.43 | -0.63 | 0.0093 |
| miR-8485 | YOD1 | 2.59 | 8.06 | -0.58 | 0.0197 |
| miR-92a-3p | MPP1 | 13.49 | 12.85 | -0.52 | 0.0394 |
| miR-942-5p | FNBP1L | 3.48 | 4.90 | -0.50 | 0.0471 |

- An unpaired two-tailed t-test was used to calculate the P-value.

**Supplementary TABLE 4 |** Significantly enriched pathways and diseases for the identified correlated miRNAs in the blood of patients with TGA compared controls (adjusted P value < 0.05)

| **Diseases** | **P-value** | **P-adjusted** | **Observed** | **miRNAs** |
| --- | --- | --- | --- | --- |
| Arrhythmias, Cardiac | 0.0121 | 0.0416 | 4 | miR-125a-5p; miR-125b-5p; miR-20a-5p; miR-30b-5p |
| Cardiomegaly | 0.0115 | 0.0416 | 7 | miR-125b-5p; miR-181b-5p; miR-19a-3p; miR-20a-5p; miR-30b-5p; miR-30c-5p; miR-92a-3p |
| Heart Failure | 0.0009 | 0.0336 | 6 | miR-125a-5p; miR-125b-5p; miR-181b-5p; miR-19a-3p; miR-20a-5p; miR-30b-5p |
| Myocardial infarction | 0.0022 | 0.0348 | 5 | miR-125b-5p; miR-181b-5p; miR-197-3p; miR-19a-3p; miR-20a-5p |
| Myocarditis | 0.0121 | 0.0416 | 4 | miR-125a-5p; miR-125b-5p; miR-20a-5p; miR-30b-5p |
|  |  |  |  |  |
| **Tissue Atlas** | **P-value** | **P-adjusted** | **Observed** | **miRNAs** |
| Myocardium | 0.0084 | 0.0109 | 27 | miR-1246; miR-125a-5p; miR-125b-5p; miR-1268b; miR-1275; miR-1305; miR-144-5p; miR-181b-5p; miR-197-3p; miR-19a-3p; miR-20a-5p; miR-30b-5p; miR-30c-5p; miR-3198; miR-33b-3p; miR-361-3p; miR-4324; miR-4428; miR-4672; miR-484; miR-5581-5p; miR-574-5p; miR-6131; miR-629-3p; miR-6511b-3p; miR-664a-3p; miR-92a-3p |
|  |  |  |  |  |
| **Pathways** | **P-value** | **P-adjusted** | **Observed** | **miRNAs** |
| Wnt signaling pathway | 0.0015 | 0.0298 | 13 | miR-125a-5p; miR-125b-5p; miR-130b-5p; miR-17-3p; miR-181b-5p; miR-197-3p; miR-19a-3p; miR-20a-5p; miR-30b-5p; miR-30c-5p; miR-361-3p; miR-484; miR-92a-3p |
| TGF beta signaling pathway | 0.0013 | 0.0289 | 11 | miR-125a-5p; miR-125b-5p; miR-130b-5p; miR-181b-5p; miR-197-3p; miR-19a-3p; miR-20a-5p; miR-30b-5p; miR-30c-5p; miR-484; miR-92a-3p |
| PDGF signaling pathway | 0.0058 | 0.0413 | 10 | miR-125a-5p; miR-125b-5p; miR-17-3p; miR-197-3p; miR-19a-3p; miR-20a-5p; miR-30b-5p; miR-30c-5p; miR-484; miR-92a-3p |
| p53 signaling pathway | 0.0050 | 0.0413 | 12 | miR-125a-5p; miR-125b-5p; miR-17-3p; miR-181b-5p; miR-197-3p; miR-19a-3p; miR-20a-5p; miR-30b-5p; miR-30c-5p; miR-484; miR-92a-3p; miR-942-5p |
| p38 MAPK Signaling Pathway | 0.0042 | 0.0413 | 8 | miR-125a-5p; miR-125b-5p; miR-197-3p; miR-19a-3p; miR-20a-5p; miR-30b-5p; miR-30c-5p; miR-484 |
| Notch signaling pathway | 0.0058 | 0.0413 | 10 | miR-125b-5p; miR-130b-5p; miR-17-3p; miR-181b-5p; miR-197-3p; miR-19a-3p; miR-20a-5p; miR-30c-5p; miR-484; miR-92a-3p |
| MAPK signaling pathway | 0.0013 | 0.0289 | 11 | miR-125a-5p; miR-125b-5p; miR-130b-5p; miR-181b-5p; miR-197-3p; miR-19a-3p; miR-20a-5p; miR-30b-5p; miR-30c-5p; miR-484; miR-92a-3p |
| Jak STAT signaling pathway | 0.0058 | 0.0413 | 10 | miR-125a-5p; miR-125b-5p; miR-130b-5p; miR-181b-5p; miR-19a-3p; miR-20a-5p; miR-30b-5p; miR-30c-5p; miR-484; miR-92a-3p |
| Insulin signaling pathway | 0.0050 | 0.0413 | 12 | miR-125a-5p; miR-125b-5p; miR-130b-5p; miR-181b-5p; miR-197-3p; miR-19a-3p; miR-20a-5p; miR-30b-5p; miR-30c-5p; miR-484; miR-92a-3p; miR-942-5p |
| FGF signaling pathway | 0.0013 | 0.0289 | 11 | miR-125a-5p; miR-125b-5p; miR-130b-5p; miR-17-3p; miR-181b-5p; miR-197-3p; miR-20a-5p; miR-30b-5p; miR-30c-5p; miR-484; miR-92a-3p |
| EGF receptor signaling pathway | 0.0058 | 0.0413 | 10 | miR-125a-5p; miR-125b-5p; miR-17-3p; miR-197-3p; miR-19a-3p; miR-20a-5p; miR-30b-5p; miR-30c-5p; miR-484; miR-92a-3p |
| EGF EGFR Signaling Pathway | 0.0013 | 0.0289 | 11 | miR-125a-5p; miR-125b-5p; miR-130b-5p; miR-17-3p; miR-181b-5p; miR-19a-3p; miR-20a-5p; miR-30b-5p; miR-30c-5p; miR-484; miR-92a-3p |
| Apoptosis signaling pathway | 0.0013 | 0.0289 | 11 | miR-125a-5p; miR-125b-5p; miR-130b-5p; miR-17-3p; miR-181b-5p; miR-197-3p; miR-19a-3p; miR-20a-5p; miR-30c-5p; miR-484; miR-92a-3p |

**SUPPLEMENTARY TABLE 5 |** Significantly abundant mRNAs in the blood of TGA patients with and without overt heart failure

| **mRNA** | **Mean OHF** | **Mean Non-OHF** | **Difference** | **Fold Change** | **Regulation** | **P value** | **Adjusted P value** |
| --- | --- | --- | --- | --- | --- | --- | --- |
| ATAD3B | 9.08 | 9.84 | 0.76 | 0.59 | Lower | 2.61E-08 | 1.78E-05 |
| BCAS4 | 4.47 | 5.21 | 0.74 | 0.60 | Lower | 1.70E-05 | 3.72E-03 |
| CFAP298 | 8.59 | 9.18 | 0.59 | 0.66 | Lower | 4.94E-07 | 1.99E-04 |
| CMTM8 | 5.99 | 7.19 | 1.20 | 0.43 | Lower | 3.25E-04 | 3.43E-02 |
| COL18A1 | 9.52 | 10.20 | 0.67 | 0.63 | Lower | 1.95E-06 | 6.27E-04 |
| EXOC7 | 8.10 | 8.74 | 0.63 | 0.65 | Lower | 6.85E-06 | 1.79E-03 |
| GAL3ST4 | 3.19 | 3.89 | 0.69 | 0.62 | Lower | 4.95E-04 | 4.43E-02 |
| GAMT | 4.73 | 5.72 | 0.99 | 0.50 | Lower | 4.30E-04 | 4.05E-02 |
| GPATCH11 | 6.58 | 7.21 | 0.64 | 0.64 | Lower | 1.34E-04 | 1.84E-02 |
| HAUS5 | 5.35 | 6.34 | 0.99 | 0.50 | Lower | 2.51E-04 | 2.89E-02 |
| HMOX2 | 9.81 | 10.45 | 0.64 | 0.64 | Lower | 2.02E-05 | 4.20E-03 |
| ID3 | 7.09 | 7.75 | 0.66 | 0.63 | Lower | 1.40E-05 | 3.18E-03 |
| JADE2 | 5.83 | 6.54 | 0.70 | 0.61 | Lower | 4.00E-04 | 3.93E-02 |
| KLHL34 | 4.35 | 5.36 | 1.01 | 0.50 | Lower | 1.02E-05 | 2.41E-03 |
| KRT72 | 5.09 | 6.69 | 1.61 | 0.33 | Lower | 3.58E-05 | 6.74E-03 |
| LMTK3 | 4.96 | 6.07 | 1.12 | 0.46 | Lower | 2.30E-12 | 5.11E-09 |
| MAP4K1 | 7.64 | 8.45 | 0.81 | 0.57 | Lower | 3.48E-04 | 3.61E-02 |
| MEI1 | 9.58 | 10.48 | 0.90 | 0.54 | Lower | 6.61E-05 | 1.06E-02 |
| MPI | 7.43 | 8.25 | 0.82 | 0.57 | Lower | 3.00E-04 | 3.26E-02 |
| MZF1 | 8.63 | 9.29 | 0.66 | 0.63 | Lower | 2.10E-04 | 2.51E-02 |
| NPM3 | 6.31 | 7.15 | 0.85 | 0.56 | Lower | 5.31E-13 | 1.34E-09 |
| OBSCN | 4.56 | 5.18 | 0.62 | 0.65 | Lower | 6.62E-06 | 1.75E-03 |
| OCIAD2 | 8.57 | 9.55 | 0.98 | 0.51 | Lower | 4.37E-05 | 8.07E-03 |
| OVGP1 | 4.96 | 5.89 | 0.93 | 0.53 | Lower | 1.40E-07 | 7.10E-05 |
| PHLDB3 | 6.49 | 7.56 | 1.08 | 0.47 | Lower | 2.53E-18 | 4.48E-14 |
| RALGDS | 9.04 | 9.87 | 0.83 | 0.56 | Lower | 2.09E-04 | 2.52E-02 |
| RANBP1 | 7.69 | 8.85 | 1.16 | 0.45 | Lower | 1.14E-04 | 1.62E-02 |
| RHOH | 8.92 | 9.94 | 1.02 | 0.49 | Lower | 3.02E-05 | 5.88E-03 |
| RPL7A | 13.68 | 14.64 | 0.96 | 0.51 | Lower | 4.12E-04 | 4.01E-02 |
| RPS23 | 11.50 | 12.23 | 0.73 | 0.60 | Lower | 1.95E-10 | 2.65E-07 |
| RPS3 | 11.19 | 12.10 | 0.91 | 0.53 | Lower | 1.85E-04 | 2.29E-02 |
| RPS6 | 13.16 | 14.13 | 0.97 | 0.51 | Lower | 6.53E-06 | 1.75E-03 |
| SAMD10 | 6.67 | 7.48 | 0.80 | 0.57 | Lower | 4.21E-04 | 4.01E-02 |
| SCML4 | 8.77 | 9.80 | 1.02 | 0.49 | Lower | 6.87E-05 | 1.10E-02 |
| SH2D3A | 7.49 | 8.63 | 1.14 | 0.45 | Lower | 1.26E-06 | 4.67E-04 |
| SLC25A23 | 4.86 | 6.20 | 1.34 | 0.40 | Lower | 6.41E-05 | 1.05E-02 |
| SLC2A4RG | 10.43 | 11.28 | 0.85 | 0.56 | Lower | 3.92E-04 | 3.88E-02 |
| SPON1 | 3.45 | 4.16 | 0.71 | 0.61 | Lower | 2.87E-04 | 3.20E-02 |
| TAS2R31 | 6.05 | 6.63 | 0.58 | 0.67 | Lower | 5.60E-04 | 4.89E-02 |
| TMEM121 | 6.45 | 7.12 | 0.67 | 0.63 | Lower | 5.69E-05 | 9.61E-03 |
| TTC39C | 6.60 | 7.24 | 0.64 | 0.64 | Lower | 1.90E-04 | 2.34E-02 |
| ABHD14A | 9.34 | 10.12 | 0.78 | 0.58 | Lower | 4.35E-04 | 4.06E-02 |
| ABHD14B | 7.30 | 7.90 | 0.60 | 0.66 | Lower | 1.14E-04 | 1.63E-02 |
| AGMAT | 5.25 | 6.27 | 1.02 | 0.49 | Lower | 3.50E-10 | 4.43E-07 |
| ATAD3A | 9.25 | 9.97 | 0.72 | 0.61 | Lower | 1.74E-06 | 5.83E-04 |
| BNIP3 | 6.82 | 7.64 | 0.82 | 0.57 | Lower | 4.22E-06 | 1.23E-03 |
| BOP1 | 6.64 | 7.23 | 0.59 | 0.66 | Lower | 6.24E-12 | 1.11E-08 |
| C6orf48 | 9.46 | 10.32 | 0.86 | 0.55 | Lower | 6.58E-11 | 9.72E-08 |
| CCT3 | 9.47 | 10.12 | 0.65 | 0.64 | Lower | 1.00E-09 | 9.87E-07 |
| CD248 | 3.58 | 4.65 | 1.07 | 0.48 | Lower | 1.53E-04 | 1.99E-02 |
| CD40LG | 8.00 | 9.57 | 1.57 | 0.34 | Lower | 3.05E-04 | 3.25E-02 |
| EEF1B2 | 10.64 | 11.50 | 0.86 | 0.55 | Lower | 1.93E-04 | 2.36E-02 |
| GPR171 | 8.73 | 9.70 | 0.97 | 0.51 | Lower | 1.78E-07 | 8.50E-05 |
| GRWD1 | 7.88 | 8.57 | 0.69 | 0.62 | Lower | 1.41E-04 | 1.89E-02 |
| HYKK | 3.25 | 3.94 | 0.69 | 0.62 | Lower | 1.63E-13 | 5.77E-10 |
| ISYNA1 | 5.31 | 6.00 | 0.69 | 0.62 | Lower | 4.93E-04 | 4.44E-02 |
| MRNIP | 8.26 | 9.26 | 0.99 | 0.50 | Lower | 2.17E-04 | 2.57E-02 |
| NOP53 | 11.79 | 12.98 | 1.20 | 0.44 | Lower | 3.04E-04 | 3.27E-02 |
| OCM2 | 5.25 | 6.11 | 0.86 | 0.55 | Lower | 1.14E-08 | 8.81E-06 |
| OXLD1 | 10.11 | 10.73 | 0.61 | 0.65 | Lower | 1.60E-07 | 7.86E-05 |
| PARP1 | 7.69 | 8.36 | 0.67 | 0.63 | Lower | 2.07E-09 | 1.94E-06 |
| PIDD1 | 9.29 | 9.95 | 0.66 | 0.63 | Lower | 3.34E-04 | 3.51E-02 |
| PTPRCAP | 9.49 | 10.49 | 0.99 | 0.50 | Lower | 4.17E-04 | 4.04E-02 |
| RACK1 | 12.34 | 12.94 | 0.60 | 0.66 | Lower | 2.31E-07 | 1.05E-04 |
| RPL10A | 13.40 | 14.34 | 0.95 | 0.52 | Lower | 4.57E-04 | 4.18E-02 |
| RPL15 | 11.88 | 12.65 | 0.77 | 0.59 | Lower | 4.64E-05 | 8.38E-03 |
| RPL18 | 13.34 | 14.35 | 1.01 | 0.50 | Lower | 3.52E-05 | 6.71E-03 |
| RPL3 | 13.07 | 14.08 | 1.01 | 0.50 | Lower | 3.97E-12 | 7.82E-09 |
| RPS18 | 13.30 | 14.18 | 0.88 | 0.54 | Lower | 9.19E-05 | 1.38E-02 |
| RPS19 | 13.48 | 14.55 | 1.07 | 0.48 | Lower | 1.59E-04 | 2.03E-02 |
| RTN4R | 5.48 | 6.15 | 0.67 | 0.63 | Lower | 2.55E-05 | 5.08E-03 |
| TARBP2 | 6.25 | 6.84 | 0.58 | 0.67 | Lower | 8.43E-06 | 2.08E-03 |
| TBC1D10C | 12.03 | 13.01 | 0.98 | 0.51 | Lower | 2.01E-07 | 9.37E-05 |
| TEDC1 | 7.34 | 8.13 | 0.79 | 0.58 | Lower | 8.71E-09 | 7.02E-06 |
| ZNF444 | 9.12 | 9.85 | 0.73 | 0.60 | Lower | 3.53E-07 | 1.45E-04 |
| IFT52 | 7.94 | 7.35 | -0.60 | 1.51 | Higher | 3.67E-04 | 3.72E-02 |
| BAG6 | 13.82 | 13.18 | -0.63 | 1.55 | Higher | 6.09E-08 | 3.48E-05 |
| LACC1 | 4.14 | 3.49 | -0.65 | 1.57 | Higher | 9.55E-07 | 3.60E-04 |
| C1orf198 | 9.46 | 8.79 | -0.67 | 1.59 | Higher | 6.95E-08 | 3.85E-05 |
| PPM1A | 7.58 | 6.87 | -0.70 | 1.63 | Higher | 9.08E-05 | 1.38E-02 |
| CYBRD1 | 7.67 | 6.96 | -0.71 | 1.63 | Higher | 8.40E-09 | 7.09E-06 |
| RCOR3 | 6.77 | 6.02 | -0.75 | 1.68 | Higher | 3.02E-04 | 3.26E-02 |
| AIDA | 5.49 | 4.72 | -0.77 | 1.71 | Higher | 1.27E-05 | 2.93E-03 |
| BRD4 | 10.91 | 10.10 | -0.80 | 1.74 | Higher | 5.59E-05 | 9.53E-03 |
| SCGB3A1 | 15.57 | 14.75 | -0.83 | 1.77 | Higher | 7.26E-05 | 1.15E-02 |
| PSMF1 | 6.73 | 5.89 | -0.84 | 1.79 | Higher | 9.08E-08 | 4.88E-05 |
| IQSEC2 | 15.57 | 14.71 | -0.85 | 1.81 | Higher | 2.18E-05 | 4.43E-03 |
| MYMK | 14.33 | 13.45 | -0.88 | 1.84 | Higher | 5.14E-10 | 5.69E-07 |
| BIRC2 | 8.28 | 7.39 | -0.89 | 1.86 | Higher | 9.83E-05 | 1.45E-02 |
| GRINA | 10.14 | 9.25 | -0.89 | 1.86 | Higher | 3.75E-04 | 3.76E-02 |
| TDRG1 | 10.87 | 9.94 | -0.93 | 1.91 | Higher | 3.19E-05 | 6.14E-03 |
| CNPPD1 | 9.56 | 8.62 | -0.94 | 1.91 | Higher | 3.23E-07 | 1.39E-04 |
| TBC1D22B | 6.99 | 6.03 | -0.96 | 1.94 | Higher | 1.74E-06 | 5.93E-04 |
| TAL1 | 6.03 | 5.00 | -1.03 | 2.05 | Higher | 2.47E-14 | 1.09E-10 |
| DAP | 9.54 | 8.50 | -1.04 | 2.05 | Higher | 1.08E-04 | 1.57E-02 |
| PCTP | 10.27 | 9.15 | -1.12 | 2.17 | Higher | 2.64E-04 | 3.02E-02 |
| UBE2H | 10.45 | 9.32 | -1.14 | 2.20 | Higher | 1.37E-04 | 1.87E-02 |
| SNX3 | 11.62 | 10.48 | -1.14 | 2.20 | Higher | 1.96E-16 | 1.73E-12 |
| GUCD1 | 11.92 | 10.76 | -1.15 | 2.22 | Higher | 2.80E-04 | 3.18E-02 |
| GPR88 | 14.01 | 12.84 | -1.17 | 2.25 | Higher | 1.52E-04 | 2.00E-02 |
| PHOSPHO1 | 15.00 | 13.83 | -1.17 | 2.26 | Higher | 4.65E-05 | 8.32E-03 |
| MRC2 | 11.11 | 9.79 | -1.31 | 2.49 | Higher | 4.41E-05 | 8.05E-03 |
| ALAS2 | 16.94 | 15.60 | -1.35 | 2.54 | Higher | 8.62E-07 | 3.32E-04 |
| DCAF6 | 7.91 | 6.54 | -1.37 | 2.59 | Higher | 2.86E-04 | 3.21E-02 |
| BNIP3L | 11.48 | 9.92 | -1.56 | 2.94 | Higher | 5.55E-05 | 9.56E-03 |
| KAT2B | 8.79 | 7.20 | -1.58 | 2.99 | Higher | 9.58E-06 | 2.29E-03 |
| ADIPOR1 | 13.40 | 11.81 | -1.59 | 3.02 | Higher | 4.85E-08 | 2.96E-05 |
| RBM38 | 12.41 | 10.69 | -1.72 | 3.29 | Higher | 1.23E-08 | 9.09E-06 |
| JAZF1 | 8.60 | 6.82 | -1.78 | 3.44 | Higher | 3.60E-08 | 2.36E-05 |
| STRADB | 12.09 | 10.29 | -1.80 | 3.49 | Higher | 7.83E-05 | 1.22E-02 |
| PORCN | 13.92 | 12.10 | -1.82 | 3.54 | Higher | 2.89E-04 | 3.20E-02 |
| CTSE | 6.20 | 4.36 | -1.83 | 3.56 | Higher | 5.41E-04 | 4.80E-02 |
| EPB42 | 13.20 | 11.34 | -1.86 | 3.63 | Higher | 5.04E-05 | 8.94E-03 |
| GLRX5 | 13.86 | 11.97 | -1.89 | 3.70 | Higher | 2.93E-04 | 3.20E-02 |
| SESN3 | 8.40 | 6.49 | -1.91 | 3.76 | Higher | 1.64E-04 | 2.07E-02 |
| BCL2L1 | 10.59 | 8.55 | -2.05 | 4.13 | Higher | 5.19E-05 | 9.10E-03 |
| TRIM58 | 13.45 | 11.39 | -2.06 | 4.17 | Higher | 4.42E-04 | 4.08E-02 |
| PITHD1 | 8.39 | 6.33 | -2.06 | 4.17 | Higher | 5.51E-06 | 1.53E-03 |
| KLF1 | 12.62 | 10.53 | -2.09 | 4.27 | Higher | 2.93E-04 | 3.22E-02 |
| SLC6A9 | 7.60 | 5.41 | -2.20 | 4.58 | Higher | 1.22E-04 | 1.70E-02 |
| TNS1 | 9.93 | 7.70 | -2.23 | 4.69 | Higher | 1.44E-06 | 5.01E-04 |
| TMOD1 | 8.83 | 6.48 | -2.35 | 5.10 | Higher | 3.11E-04 | 3.30E-02 |
| FLCN | 11.54 | 9.15 | -2.39 | 5.24 | Higher | 2.48E-04 | 2.87E-02 |
| RNF182 | 7.42 | 4.51 | -2.92 | 7.54 | Higher | 5.38E-04 | 4.79E-02 |

- An unpaired two-tailed t-test was used to calculate the P-value.

**SUPPLEMENTARY TABLE 6 |** Significantly abundant mRNAs in the blood of TGA patients died due to sudden cardiac death compared with subjects without death.

| **mRNA** | **Mean Death** | **Mean Non-Death** | **Difference** | **Fold Change** | **Regulation** | **P value** | **Adjusted P value** |
| --- | --- | --- | --- | --- | --- | --- | --- |
| KCNG1 | 5.43 | 7.34 | 1.90 | 0.27 | Lower | 3.57E-05 | 9.58E-03 |
| IL4R | 11.99 | 13.29 | 1.30 | 0.41 | Lower | 1.37E-06 | 8.35E-04 |
| C12orf42 | 4.71 | 5.84 | 1.13 | 0.46 | Lower | 5.14E-08 | 5.70E-05 |
| CMTM8 | 6.09 | 7.18 | 1.10 | 0.47 | Lower | 2.22E-05 | 6.66E-03 |
| FCRL3 | 8.14 | 9.17 | 1.03 | 0.49 | Lower | 1.69E-06 | 9.37E-04 |
| TSHZ1 | 6.92 | 7.91 | 0.99 | 0.50 | Lower | 1.29E-05 | 4.65E-03 |
| FCGR3A | 14.40 | 15.38 | 0.98 | 0.51 | Lower | 3.40E-04 | 4.43E-02 |
| ADAM8 | 13.91 | 14.70 | 0.80 | 0.58 | Lower | 2.03E-04 | 3.14E-02 |
| NCAPD2 | 8.07 | 8.85 | 0.78 | 0.58 | Lower | 6.26E-07 | 4.83E-04 |
| SH2D3C | 11.44 | 12.21 | 0.77 | 0.58 | Lower | 7.01E-05 | 1.50E-02 |
| SMAD3 | 7.50 | 8.26 | 0.77 | 0.59 | Lower | 1.25E-08 | 2.01E-05 |
| ITFG2 | 6.91 | 7.66 | 0.75 | 0.60 | Lower | 3.66E-05 | 9.68E-03 |
| SRSF4 | 9.78 | 10.52 | 0.74 | 0.60 | Lower | 4.96E-08 | 5.86E-05 |
| TOB2 | 6.52 | 7.25 | 0.73 | 0.60 | Lower | 2.32E-07 | 2.29E-04 |
| ALOX5 | 12.74 | 13.47 | 0.73 | 0.60 | Lower | 5.89E-07 | 4.74E-04 |
| LSP1 | 13.31 | 14.03 | 0.72 | 0.61 | Lower | 1.38E-05 | 4.80E-03 |
| SPON1 | 3.45 | 4.16 | 0.71 | 0.61 | Lower | 2.58E-04 | 3.75E-02 |
| TSEN34 | 12.58 | 13.28 | 0.70 | 0.62 | Lower | 2.49E-05 | 7.25E-03 |
| MSN | 11.68 | 12.37 | 0.69 | 0.62 | Lower | 1.54E-04 | 2.52E-02 |
| SLC12A6 | 9.04 | 9.73 | 0.69 | 0.62 | Lower | 1.05E-04 | 1.90E-02 |
| LCP1 | 13.74 | 14.43 | 0.69 | 0.62 | Lower | 2.14E-05 | 6.65E-03 |
| MKNK2 | 12.86 | 13.55 | 0.69 | 0.62 | Lower | 3.81E-05 | 9.92E-03 |
| TTC39C | 6.56 | 7.25 | 0.68 | 0.62 | Lower | 4.31E-09 | 9.56E-06 |
| COL18A1 | 9.52 | 10.20 | 0.68 | 0.63 | Lower | 9.22E-07 | 5.83E-04 |
| RASSF2 | 12.69 | 13.36 | 0.68 | 0.63 | Lower | 1.89E-05 | 6.20E-03 |
| NDRG3 | 5.71 | 6.38 | 0.67 | 0.63 | Lower | 6.98E-05 | 1.51E-02 |
| ZC3H4 | 8.42 | 9.08 | 0.66 | 0.63 | Lower | 3.37E-06 | 1.66E-03 |
| KANSL1 | 9.23 | 9.89 | 0.66 | 0.63 | Lower | 5.89E-06 | 2.55E-03 |
| SLC6A16 | 5.79 | 6.42 | 0.63 | 0.65 | Lower | 1.69E-06 | 9.10E-04 |
| VEZF1 | 8.62 | 9.24 | 0.61 | 0.65 | Lower | 2.03E-04 | 3.15E-02 |
| LZTR1 | 6.33 | 6.94 | 0.61 | 0.65 | Lower | 1.03E-04 | 1.88E-02 |
| HTATIP2 | 4.64 | 5.25 | 0.61 | 0.66 | Lower | 3.72E-04 | 4.71E-02 |
| ARHGAP25 | 12.38 | 12.99 | 0.60 | 0.66 | Lower | 9.57E-05 | 1.82E-02 |
| TBC1D2B | 9.35 | 9.95 | 0.60 | 0.66 | Lower | 3.21E-04 | 4.24E-02 |
| FCHO1 | 10.76 | 11.36 | 0.60 | 0.66 | Lower | 2.82E-05 | 7.94E-03 |
| MED8 | 7.62 | 8.22 | 0.60 | 0.66 | Lower | 3.91E-07 | 3.46E-04 |
| IQSEC1 | 11.36 | 11.96 | 0.60 | 0.66 | Lower | 1.68E-05 | 5.62E-03 |
| PHF12 | 5.74 | 6.34 | 0.60 | 0.66 | Lower | 9.75E-11 | 5.76E-07 |
| BCL3 | 10.74 | 11.63 | 0.89 | 0.54 | Lower | 6.92E-07 | 4.72E-04 |
| THAP7 | 5.55 | 6.37 | 0.82 | 0.57 | Lower | 3.22E-04 | 4.23E-02 |
| CLSTN1 | 7.32 | 7.99 | 0.67 | 0.63 | Lower | 5.25E-05 | 1.26E-02 |
| ABHD11 | 5.93 | 5.34 | -0.59 | 1.51 | Higher | 2.61E-09 | 7.72E-06 |
| LACC1 | 4.10 | 3.49 | -0.60 | 1.52 | Higher | 6.61E-07 | 4.69E-04 |
| ATP5F1C | 7.87 | 7.25 | -0.62 | 1.54 | Higher | 1.28E-04 | 2.23E-02 |
| CRHR1 | 6.09 | 5.46 | -0.63 | 1.55 | Higher | 8.80E-05 | 1.71E-02 |
| NCCRP1 | 5.72 | 5.06 | -0.67 | 1.59 | Higher | 1.99E-09 | 7.07E-06 |
| AIDA | 5.49 | 4.72 | -0.77 | 1.70 | Higher | 1.52E-05 | 5.19E-03 |
| LHL29 | 6.66 | 5.87 | -0.79 | 1.73 | Higher | 1.40E-10 | 6.21E-07 |
| BIRC2 | 8.21 | 7.40 | -0.81 | 1.75 | Higher | 1.55E-06 | 9.14E-04 |
| NHLH1 | 4.44 | 3.58 | -0.85 | 1.81 | Higher | 4.06E-05 | 1.04E-02 |
| TAL1 | 5.96 | 5.01 | -0.95 | 1.93 | Higher | 1.34E-05 | 4.73E-03 |
| SHARPIN | 11.40 | 10.40 | -1.00 | 1.99 | Higher | 4.21E-12 | 3.73E-08 |
| GID4 | 9.22 | 8.19 | -1.02 | 2.03 | Higher | 8.89E-06 | 3.58E-03 |
| PCTP | 10.22 | 9.16 | -1.06 | 2.09 | Higher | 1.37E-04 | 2.33E-02 |
| ICAM4 | 9.19 | 8.12 | -1.07 | 2.10 | Higher | 5.81E-06 | 2.57E-03 |
| MAF1 | 9.61 | 8.51 | -1.10 | 2.15 | Higher | 6.43E-05 | 1.42E-02 |
| FHL1 | 10.83 | 9.68 | -1.15 | 2.22 | Higher | 5.21E-09 | 1.03E-05 |
| PPP1R14A | 12.30 | 11.00 | -1.29 | 2.45 | Higher | 9.32E-05 | 1.80E-02 |
| KAT2B | 8.61 | 7.22 | -1.39 | 2.61 | Higher | 1.60E-06 | 9.14E-04 |
| MCAT | 10.38 | 8.87 | -1.51 | 2.85 | Higher | 1.31E-07 | 1.37E-04 |
| RBM38 | 12.29 | 10.70 | -1.59 | 3.01 | Higher | 1.17E-05 | 4.41E-03 |
| JAZF1 | 8.48 | 6.83 | -1.66 | 3.15 | Higher | 8.08E-05 | 1.61E-02 |
| CTNNAL1 | 8.05 | 6.34 | -1.70 | 3.26 | Higher | 3.14E-04 | 4.24E-02 |
| SESN3 | 8.30 | 6.50 | -1.80 | 3.49 | Higher | 4.64E-05 | 1.16E-02 |
| MARCH2 | 10.03 | 7.98 | -2.05 | 4.14 | Higher | 1.18E-14 | 2.08E-10 |
| SPTB | 6.57 | 4.40 | -2.17 | 4.50 | Higher | 4.12E-04 | 4.97E-02 |
| A4GALT | 8.15 | 5.83 | -2.31 | 4.97 | Higher | 1.78E-04 | 2.83E-02 |
| RPS4Y1 | 10.22 | 7.88 | -2.33 | 5.04 | Higher | 4.17E-04 | 4.99E-02 |
| RNF182 | 7.60 | 4.49 | -3.11 | 8.61 | Higher | 6.81E-05 | 1.49E-02 |

- An unpaired two-tailed t-test was used to calculate the P-value.

**SUPPLEMENTARY TABLE 7 |** Diagnostic power of miRNA and heart failure marker combination in TGA-RV patients compared to TGA-LV.

| **NT‐ProBNP/miRNA** | **AUC** | **Adjusted P-value** |  | **high‐sensitivity troponin T/miRNA** | **AUC** | **Adjusted P-value** |  | **eGFR /miRNA** | **AUC** | **Adjusted P-value** |
| --- | --- | --- | --- | --- | --- | --- | --- | --- | --- | --- |
| NT‐ProBNP | 0.68 | 0.23025 |  | high‐sensitivity troponin T | 0.62 | 0.14343 |  | eGFR | 0.59 | 0.16519 |
| hsa-miR-454-5p | 0.87 | 0.00240 |  | hsa-miR-6794-3p | 1.00 | 0.00240 |  | hsa-miR-454-5p | 0.87 | 0.00240 |
| hsa-miR-3135b | 0.92 | 0.00240 |  | hsa-miR-454-5p | 0.88 | 0.00240 |  | hsa-miR-3135b | 0.92 | 0.00650 |
| hsa-miR-6794-3p | 0.93 | 0.00242 |  | hsa-miR-3135b | 0.98 | 0.00240 |  | hsa-miR-6794-3p | 0.89 | 0.00661 |
| hsa-miR-1246 | 0.91 | 0.00627 |  | hsa-miR-1246 | 0.98 | 0.00397 |  | hsa-miR-22-5p | 0.83 | 0.00885 |
| hsa-miR-652-3p | 0.89 | 0.00661 |  | hsa-miR-3200-3p | 0.89 | 0.00627 |  | hsa-miR-1246 | 0.96 | 0.00885 |
| hsa-miR-145-5p | 0.87 | 0.00661 |  | hsa-miR-145-5p | 0.87 | 0.00661 |  | hsa-miR-4449 | 0.87 | 0.00963 |
| hsa-miR-3200-3p | 0.87 | 0.00823 |  | hsa-miR-193b-3p | 0.91 | 0.00661 |  | hsa-miR-145-5p | 0.84 | 0.01067 |
| hsa-miR-4449 | 0.91 | 0.00823 |  | hsa-miR-652-3p | 0.90 | 0.00823 |  | hsa-miR-4324 | 0.81 | 0.01152 |
| hsa-miR-22-5p | 0.87 | 0.00853 |  | hsa-miR-22-5p | 0.93 | 0.00823 |  | hsa-miR-193b-3p | 0.82 | 0.01184 |
| hsa-miR-193b-3p | 0.84 | 0.00867 |  | hsa-miR-4449 | 0.96 | 0.00823 |  | hsa-miR-629-3p | 0.78 | 0.01196 |
| hsa-miR-942-5p | 0.78 | 0.00867 |  | hsa-miR-942-5p | 0.78 | 0.00848 |  | hsa-miR-574-3p | 0.91 | 0.01245 |
| hsa-miR-629-3p | 0.80 | 0.00867 |  | hsa-miR-296-5p | 0.80 | 0.00885 |  | hsa-miR-3200-3p | 0.80 | 0.01318 |
| hsa-miR-4324 | 0.89 | 0.01006 |  | hsa-miR-629-3p | 0.89 | 0.00903 |  | hsa-miR-93-3p | 0.83 | 0.01319 |
| hsa-miR-296-5p | 0.84 | 0.01184 |  | hsa-miR-4324 | 0.87 | 0.01088 |  | hsa-miR-942-5p | 0.76 | 0.01429 |
| hsa-miR-574-3p | 0.93 | 0.01408 |  | hsa-miR-3605-3p | 0.84 | 0.01107 |  | hsa-miR-130b-5p | 0.78 | 0.01429 |
| hsa-miR-130b-5p | 0.87 | 0.01408 |  | hsa-miR-130b-5p | 0.84 | 0.01187 |  | hsa-miR-505-3p | 0.71 | 0.02139 |
| hsa-miR-6803-3p | 0.88 | 0.01417 |  | hsa-miR-574-3p | 0.93 | 0.01200 |  | hsa-miR-6803-3p | 0.77 | 0.02209 |
| hsa-miR-3605-3p | 0.76 | 0.01429 |  | hsa-miR-1306-5p | 0.92 | 0.01318 |  | hsa-miR-3605-3p | 0.76 | 0.02387 |
| hsa-miR-664a-3p | 0.84 | 0.01453 |  | hsa-miR-6803-3p | 0.88 | 0.01403 |  | hsa-miR-652-3p | 0.73 | 0.02858 |
| hsa-miR-93-3p | 0.84 | 0.01510 |  | hsa-miR-26b-3p | 0.89 | 0.01408 |  | hsa-miR-296-5p | 0.80 | 0.02968 |
| hsa-miR-26b-3p | 0.82 | 0.01725 |  | hsa-miR-93-3p | 0.88 | 0.01429 |  | hsa-miR-339-5p | 0.73 | 0.03066 |
| hsa-miR-1306-5p | 0.89 | 0.01733 |  | hsa-miR-339-5p | 0.78 | 0.01460 |  | hsa-miR-1306-5p | 0.87 | 0.03087 |
| hsa-miR-339-5p | 0.83 | 0.01768 |  | hsa-miR-3940-3p | 0.80 | 0.02111 |  | hsa-miR-26b-3p | 0.78 | 0.03087 |
| hsa-miR-181b-5p | 0.83 | 0.02174 |  | hsa-miR-181b-5p | 0.83 | 0.02127 |  | hsa-miR-125b-5p | 0.81 | 0.03153 |
| hsa-miR-99b-5p | 0.78 | 0.02432 |  | hsa-miR-4732-5p | 0.74 | 0.02209 |  | hsa-miR-181b-5p | 0.83 | 0.03183 |
| hsa-miR-125b-5p | 0.76 | 0.02838 |  | hsa-miR-664a-3p | 0.84 | 0.02216 |  | hsa-miR-664a-3p | 0.80 | 0.03401 |
| hsa-miR-3940-3p | 0.78 | 0.02891 |  | hsa-miR-8485 | 0.86 | 0.02244 |  | hsa-miR-99b-5p | 0.71 | 0.03654 |
| hsa-miR-4732-5p | 0.78 | 0.02920 |  | hsa-miR-99b-5p | 0.82 | 0.02283 |  | hsa-miR-3940-3p | 0.73 | 0.03740 |
| hsa-miR-8485 | 0.82 | 0.02997 |  | hsa-miR-4433a-5p | 0.88 | 0.02581 |  | hsa-miR-186-5p | 0.73 | 0.03862 |
| hsa-miR-4672 | 0.84 | 0.03078 |  | hsa-miR-212-3p | 0.82 | 0.02651 |  | hsa-miR-181a-2-3p | 0.72 | 0.03903 |
| hsa-miR-505-3p | 0.78 | 0.03237 |  | hsa-miR-4672 | 0.88 | 0.02709 |  | hsa-miR-5690 | 0.69 | 0.04086 |
| hsa-miR-2861 | 0.73 | 0.03524 |  | hsa-miR-6865-3p | 0.78 | 0.02891 |  | hsa-miR-4433a-5p | 0.83 | 0.04103 |
| hsa-miR-4433a-5p | 0.81 | 0.03701 |  | hsa-miR-2861 | 0.76 | 0.02931 |  | hsa-miR-6865-3p | 0.71 | 0.04177 |
| hsa-miR-212-3p | 0.87 | 0.03701 |  | hsa-miR-3196 | 0.86 | 0.02963 |  | hsa-miR-4732-5p | 0.73 | 0.04349 |
| hsa-miR-33b-3p | 0.74 | 0.03740 |  | hsa-miR-33b-3p | 0.80 | 0.02970 |  | hsa-miR-33b-3p | 0.76 | 0.04598 |
| hsa-miR-6865-3p | 0.72 | 0.03903 |  | hsa-miR-505-3p | 0.74 | 0.03041 |  | hsa-miR-361-5p | 0.69 | 0.05233 |
| hsa-miR-186-5p | 0.76 | 0.03958 |  | hsa-miR-186-5p | 0.78 | 0.03231 |  | hsa-miR-212-3p | 0.82 | 0.05268 |
| hsa-miR-6766-3p | 0.84 | 0.04318 |  | hsa-miR-125b-5p | 0.76 | 0.03305 |  | hsa-miR-6766-3p | 0.76 | 0.05429 |
| hsa-miR-5690 | 0.76 | 0.04431 |  | hsa-miR-6766-3p | 0.90 | 0.03401 |  | hsa-miR-4672 | 0.74 | 0.05654 |
| hsa-miR-874-3p | 0.84 | 0.04603 |  | hsa-miR-5690 | 0.83 | 0.03401 |  | hsa-miR-8485 | 0.73 | 0.05696 |
| hsa-miR-409-3p | 0.67 | 0.04622 |  | hsa-miR-4428 | 0.83 | 0.03524 |  | hsa-miR-3196 | 0.75 | 0.05782 |
| hsa-miR-3196 | 0.79 | 0.04701 |  | hsa-miR-409-3p | 0.73 | 0.03561 |  | hsa-miR-125a-5p | 0.69 | 0.05804 |
| hsa-miR-4428 | 0.81 | 0.04742 |  | hsa-miR-874-3p | 0.89 | 0.03744 |  | hsa-miR-6717-5p | 0.81 | 0.05850 |
| hsa-miR-181a-2-3p | 0.76 | 0.04908 |  | hsa-miR-181a-2-3p | 0.83 | 0.03958 |  | hsa-miR-409-3p | 0.64 | 0.06019 |
| hsa-miR-6511b-3p | 0.71 | 0.05136 |  | hsa-miR-6511b-3p | 0.78 | 0.03958 |  | hsa-miR-378a-5p | 0.63 | 0.06703 |
| hsa-miR-125a-5p | 0.69 | 0.05825 |  | hsa-miR-1271-5p | 0.82 | 0.04448 |  | hsa-miR-874-3p | 0.76 | 0.06878 |

- A receiver operating characteristic (ROC) curve analysis was performed, and the area under the ROC curve (AUC) was calculated to evaluate the diagnostic value. Adjusted P-values calculated based on the F-test on logistic regression models trained with the corresponding variables, with a Benjamini Hochberg Multiple Comparison Test (FDR = 0.05).
