# Supplementary figures and images for: Expression profiling analysis reveals key microRNA–mRNA interactions in patients with transposition of the great arteries and systemic left and right ventricles
Source: Front Cardiovasc Med. 2023 Jan 12;9:1056427. doi: 10.3389/fcvm.2022.1056427 (PMC9878113; doi:10.3389/fcvm.2022.1056427)

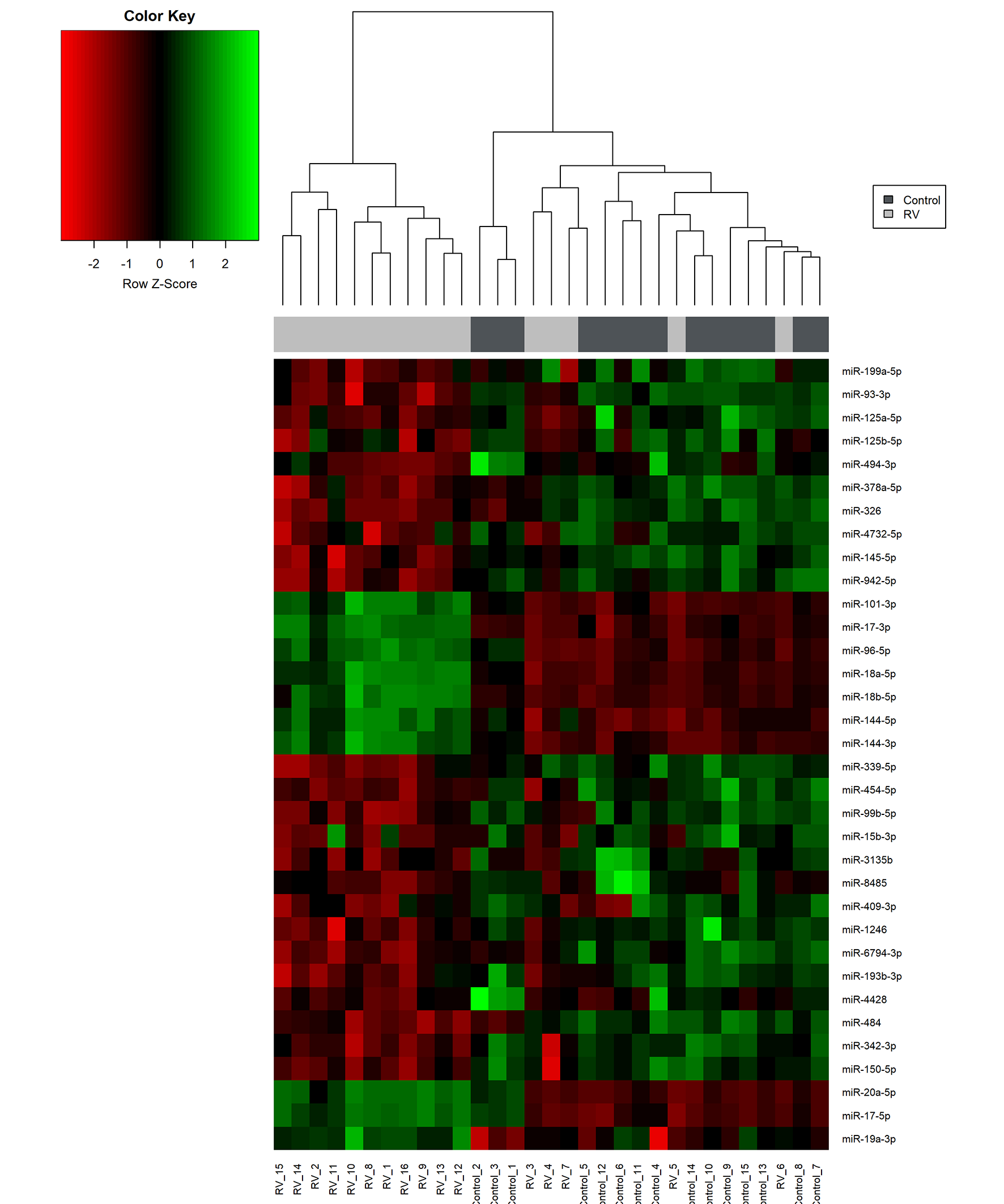

Supplement: Supplementary Figure 1 — Unsupervised hierarchical clustering (Euclidian distance, complete linkage) of the patients with TGA-RV and controls based on the differentially expressed miRNAs with the significant highest variance. [file Image_1.TIFF]

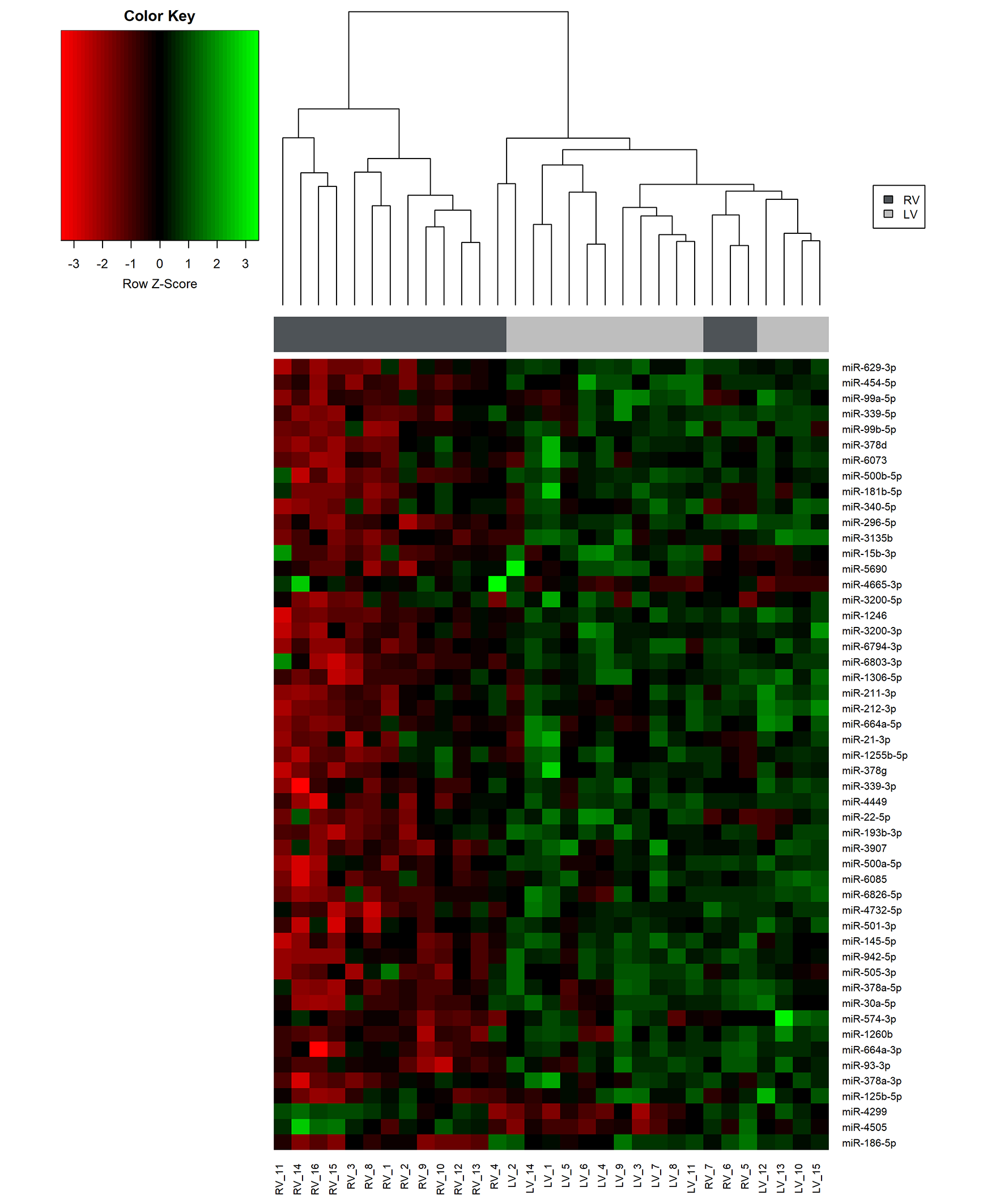

Supplement: Supplementary Figure 2 — Unsupervised hierarchical clustering (Euclidian distance, complete linkage) of the patients with TGA-RV and TGA-LV based on the differentially expressed miRNAs with the significant highest variance. [file Image_2.TIFF]

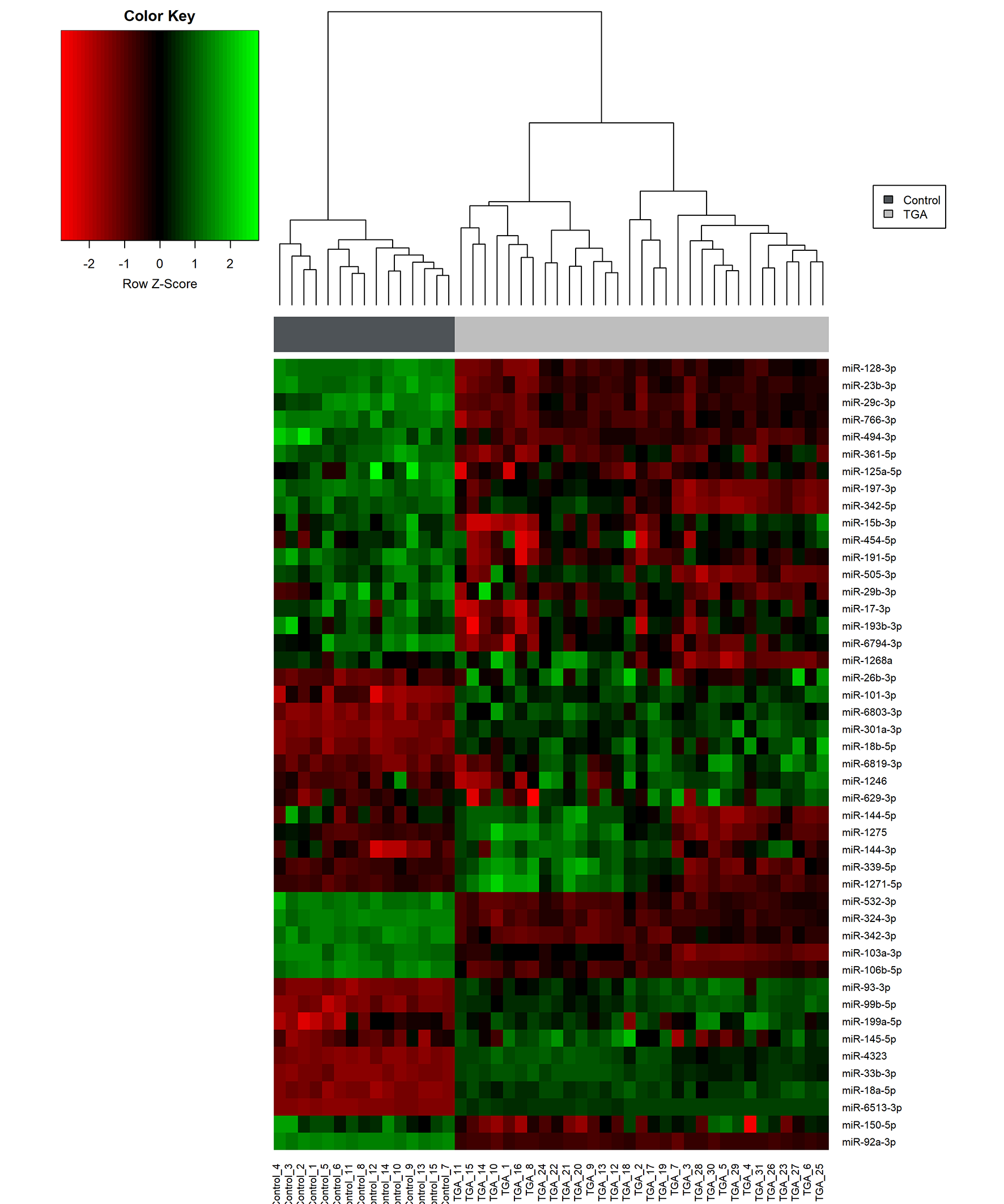

Supplement: Supplementary Figure 3 — Unsupervised hierarchical clustering (Euclidian distance, complete linkage) of the patients with TGA and controls based on the differentially expressed miRNAs with the significant highest variance. [file Image_3.TIFF]

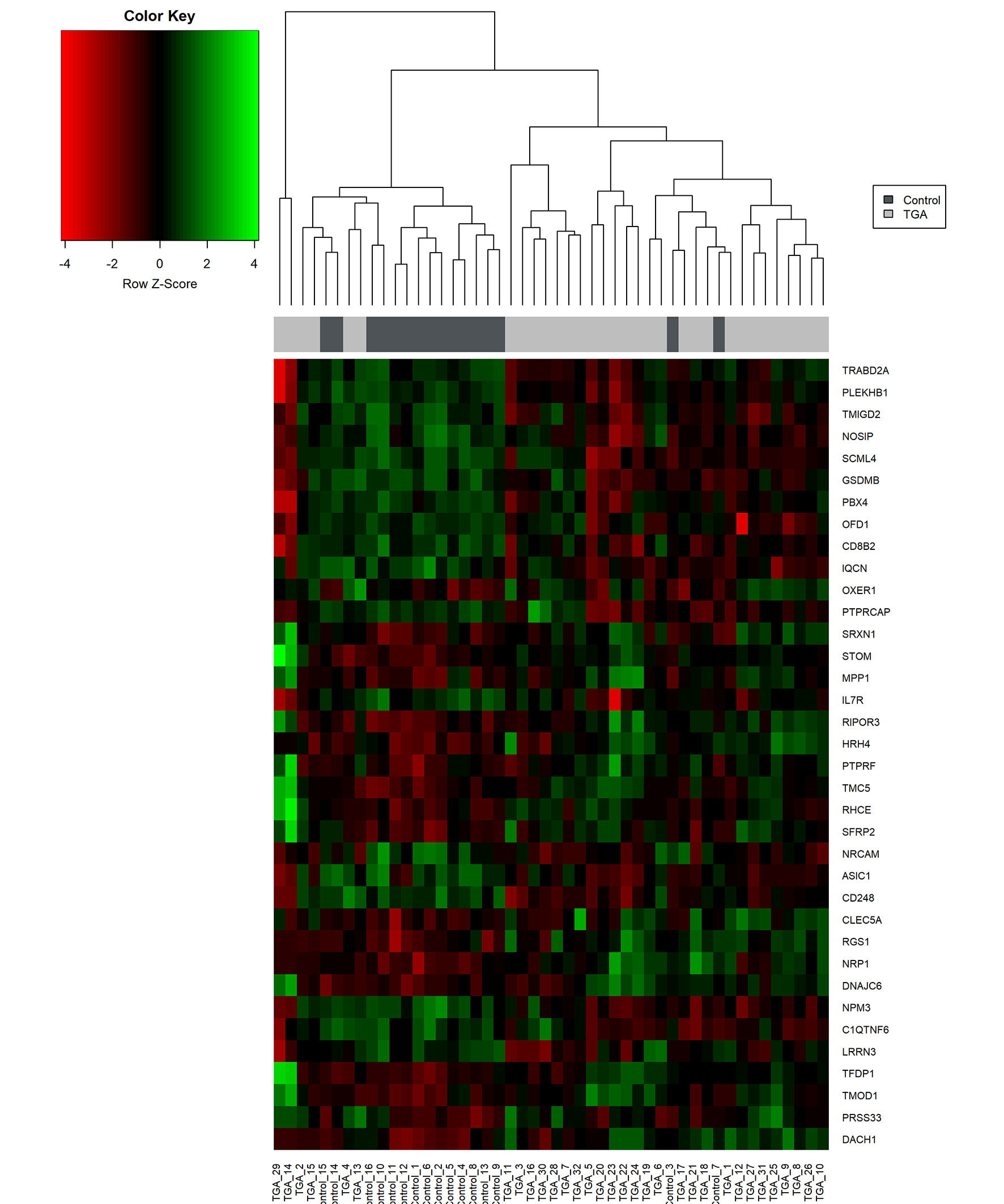

Supplement: Supplementary Figure 4 — Unsupervised hierarchical clustering (Euclidian distance, complete linkage) of the patients with TGA and controls based on the differentially expressed mRNAs with the significant highest variance. [file Image_4.TIFF]

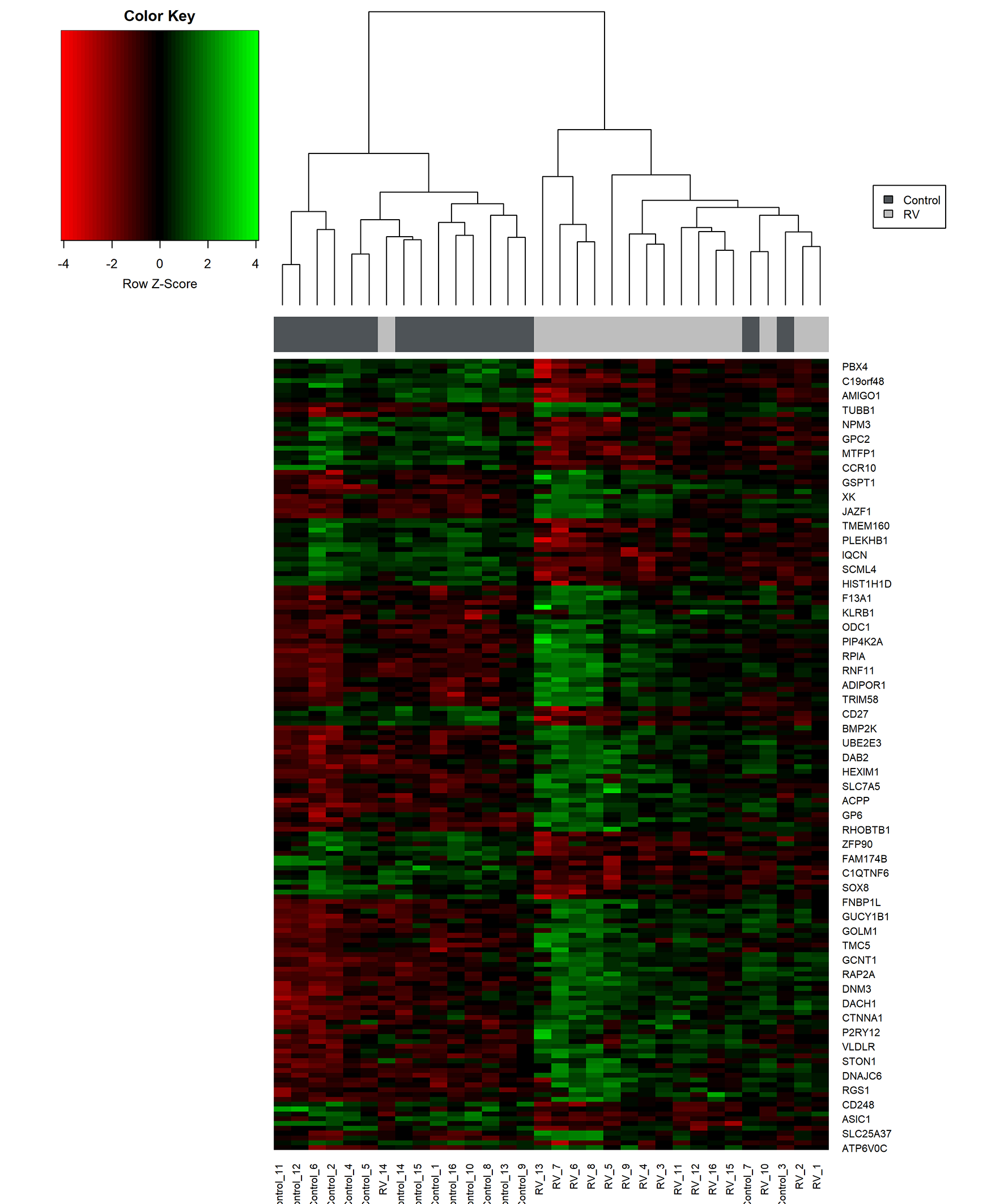

Supplement: Supplementary Figure 5 — Unsupervised hierarchical clustering (Euclidian distance, complete linkage) of the patients with TGA-RV and controls based on the differentially expressed mRNAs with the significant highest variance. [file Image_5.TIFF]
